# Combined Antioxidant Effects of Kaempferol and N-Acetylcysteine on H_2_O_2_-Induced Oxidative Stress in SH-SY5Y Cells

**DOI:** 10.3390/foods15213865

**Published:** 2026-10-28

**Authors:** Nut Palachai, Nootchanat Mairuae, Yutthana Chotritthirong

**Affiliations:** 1 Faculty of Medicine, Mahasarakham University, Maha Sarakham 44000, Thailand; nootchanat.m@msu.ac.th; 2 Graduate School of Faculty of Pharmaceutical Sciences, Khon Kaen University, Khon Kaen 40002, Thailand; yuttana.cho@nmu.ac.th

**Keywords:** kaempferol, N-acetylcysteine, oxidative stress, antioxidant, sirtuin, FOXO3a, SOD2, synergy

## Abstract

This study investigated the combined antioxidant effects of kaempferol (KAE) and N-acetylcysteine (NAC) against hydrogen peroxide (H_2_O_2_)-induced oxidative stress in SH-SY5Y cells. Combination effects were evaluated using combination index (CI) analysis and SynergyFinder. Cell viability, 5-(and-6)-chloromethyl-2′,7′-dichlorodihydrofluorescein diacetate (CM-H_2_DCFDA) fluorescence, malondialdehyde (MDA), antioxidant enzyme activities, glutathione status, and protein expression of sirtuin 3 (SIRT3), forkhead box O3 (FOXO3a), and superoxide dismutase 2 (SOD2) were assessed. Molecular docking was performed to predict potential interactions of KAE and NAC with SIRT3. The strongest CI-defined synergy occurred at 16 μM KAE and 2 mM NAC (CI = 0.10), whereas SynergyFinder results varied across reference models. The combination (KNAC) enhanced cell viability, reduced CM-H_2_DCFDA fluorescence and MDA levels, improved antioxidant enzyme activities and glutathione redox balance, and increased SIRT3, FOXO3a, and SOD2 protein levels compared with individual treatments. Molecular docking predicted a more favorable binding score for KAE than NAC against SIRT3. These findings suggest that KAE and NAC may produce complementary antioxidant and cytoprotective effects through potentially distinct mechanisms, with KAE potentially contributing to changes in proteins associated with mitochondrial antioxidant defense and NAC supporting glutathione-dependent cellular redox homeostasis. However, the causal involvement of the SIRT3/FOXO3a/SOD2 pathway and direct interactions with SIRT3 remain to be experimentally confirmed. These preliminary findings support further investigation of combining food-derived KAE with NAC as a glutathione precursor to enhance cellular antioxidant defenses.

## 1. Introduction

Oxidative stress is closely involved in cellular dysfunction and neuronal injury and develops when the production of reactive oxygen species (ROS) exceeds the capacity of cellular antioxidant defenses [1]. The brain is particularly vulnerable to oxidative damage because of its high metabolic activity, substantial oxygen consumption, abundance of polyunsaturated fatty acids, and relatively limited antioxidant capacity [2]. Excessive ROS can promote lipid peroxidation, protein and DNA damage, mitochondrial dysfunction, and disruption of cellular redox homeostasis, which may ultimately compromise neuronal survival [3,4]. Oxidative stress has also been implicated in the pathogenesis of several neurodegenerative disorders, including Alzheimer’s disease and Parkinson’s disease [5]. Since oxidative damage can accumulate over time and may precede the appearance of clinical symptoms, maintaining endogenous antioxidant defenses and cellular redox balance has received increasing attention as a potential approach to limiting oxidative stress-related neuronal damage.

Kaempferol (KAE) is a naturally occurring flavonol found in a variety of fruits, vegetables, tea, and other plant-derived foods. It has been investigated for its antioxidant, anti-inflammatory, and cytoprotective activities in several experimental models [6,7]. The antioxidant effects of KAE are not limited to direct scavenging of reactive species; KAE can also influence cellular antioxidant systems and pathways involved in the maintenance of redox homeostasis [8]. One pathway of particular interest involves sirtuin 3 (SIRT3), a mitochondrial NAD^+^-dependent deacetylase that contributes to the regulation of mitochondrial function and antioxidant defense. SIRT3 has been linked to the regulation of forkhead box O3 (FOXO3a) and mitochondrial superoxide dismutase 2 (SOD2), both of which are important components of cellular protection against oxidative stress [9]. Thus, modulation of the SIRT3/FOXO3a/SOD2 axis may represent one mechanism by which KAE contributes to the maintenance of mitochondrial redox homeostasis.

N-acetylcysteine (NAC) is a thiol-containing derivative of L-cysteine and an established precursor for intracellular glutathione (GSH) synthesis [10]. Its antioxidant activity is largely mediated by increasing intracellular cysteine availability, thereby supporting GSH synthesis and cellular thiol redox homeostasis. GSH is an important intracellular reducing agent and provides reducing equivalents for glutathione peroxidases (GSH-Px), which reduce hydrogen peroxide (H2O2) and lipid hydroperoxides to less reactive products [11]. In this regard, the antioxidant action of NAC differs from that of KAE. Whereas KAE may influence mitochondrial antioxidant signaling, NAC primarily supports cellular redox buffering through the cysteine–GSH system. The two compounds may therefore act at complementary levels of the antioxidant defense network rather than through a single shared pathway.

Combining antioxidants with different mechanisms of action may provide greater protection than increasing the concentration of a single compound [12]. Oxidative stress involves several interconnected processes, and reinforcement of one antioxidant system may not fully compensate for dysfunction in another. A combination of KAE and NAC could, in principle, provide simultaneous support for mitochondrial antioxidant defense and glutathione-dependent redox regulation. Such complementary activity may be particularly relevant under conditions of marked oxidative stress, where both mitochondrial ROS control and intracellular reducing capacity are challenged. However, whether KAE and NAC interact synergistically across different concentration combinations and the mechanisms potentially underlying their interaction have yet to be established.

In the present study, we investigated the effects of kaempferol (KAE), N-acetylcysteine (NAC), and their combination (KNAC) in an H_2_O_2_-induced oxidative stress model using SH-SY5Y neuroblastoma cells. Cell viability, intracellular oxidant-sensitive fluorescence assessed using 5-(and-6)-chloromethyl-2′,7′-dichlorodihydrofluorescein diacetate (CM-H_2_DCFDA), malondialdehyde (MDA) levels, and the activities of superoxide dismutase (SOD), catalase (CAT), and glutathione peroxidase (GSH-Px) were measured to characterize oxidative injury and cellular antioxidant responses. The interaction between KAE and NAC was evaluated using combination index (CI) analysis and SynergyFinder. We also examined glutathione status and the protein expression of sirtuin 3 (SIRT3), forkhead box O3 (FOXO3a), and superoxide dismutase 2 (SOD2) to investigate changes in cellular redox homeostasis and proteins associated with mitochondrial antioxidant defense. Molecular docking was performed to predict potential interactions of KAE and NAC with SIRT3.

We hypothesized that KAE and NAC would produce complementary antioxidant and cytoprotective effects through potentially distinct mechanisms, with KAE contributing to changes in the expression of proteins associated with mitochondrial antioxidant defense, including SIRT3, FOXO3a, and SOD2, and NAC supporting GSH-dependent cellular redox defense. The findings were expected to provide preliminary evidence regarding the potential of combining food-derived KAE with NAC to enhance cellular antioxidant defenses, while identifying potential molecular associations for further investigation.

## 2. Materials and Methods

### 2.1. Chemicals and Reagents

Kaempferol (KAE; purity 99.92%; CAS No. 520-18-3; molecular formula C_15_H_10_O_6_; molecular weight 286.24 g/mol) was purchased from MedChemExpress (Monmouth Junction, NJ, USA). N-acetylcysteine (NAC; purity 99.86%; CAS No. 616-91-1; molecular formula C_5_H_9_NO_3_S; molecular weight 163.20 g/mol) was obtained from MedChemExpress. 3-(4,5-Dimethylthiazol-2-yl)-2,5-diphenyltetrazolium bromide (MTT) and dimethyl sulfoxide (DMSO) were purchased from Merck KGaA (Darmstadt, Germany). Dulbecco’s Modified Eagle Medium (DMEM), fetal bovine serum (FBS), penicillin–streptomycin, non-essential amino acids (NEAA), and CM-H_2_DCFDA [5-(and-6)-chloromethyl-2′,7′-dichlorodihydrofluorescein diacetate] were obtained from Thermo Fisher Scientific (Waltham, MA, USA).

Reagents used for the determination of oxidative stress markers and antioxidant enzyme activities included thiobarbituric acid (TBA), potassium permanganate (KMnO_4_), cytochrome c, xanthine, xanthine oxidase (from bovine milk), dithiothreitol (DTT), sodium dodecyl sulfate (SDS), acetic acid (CH_3_COOH), ethylenediaminetetraacetic acid (EDTA), sodium azide (NaN_3_), and 1,1,3,3-tetramethoxypropane (TMP), all obtained from MilliporeSigma (Burlington, MA, USA). Potassium dihydrogen phosphate (KH_2_PO_4_), hydrogen peroxide (H_2_O_2_; 30% *w*/*w*), and sulfuric acid (H_2_SO_4_) were purchased from Merck KGaA.

Intracellular glutathione levels were determined using a Glutathione Assay Kit (Cat. No. 703002; Cayman Chemical, Ann Arbor, MI, USA). The assay was used to determine reduced glutathione (GSH), oxidized glutathione (GSSG), and total glutathione levels, with the GSH/GSSG ratio calculated as an indicator of cellular redox status.

For Western blot analysis, nitrocellulose membranes, SDS-polyacrylamide gels, and Tween-20 were obtained from Bio-Rad Laboratories (Hercules, CA, USA). Primary antibodies against sirtuin 3 (SIRT3; C73E3, Cat. No. 2627S), forkhead box O3 (FOXO3a; D19A7, Cat. No. 12829S), and superoxide dismutase 2 (SOD2; D3 × 8F, Cat. No. 13141S), together with β-actin as the loading control (13E5, Cat. No. 4970S), were obtained from Cell Signaling Technology (Danvers, MA, USA). The HRP-linked anti-rabbit IgG secondary antibody (Cat. No. 7074S) was also obtained from Cell Signaling Technology. The Immobilon^®^ Forte Western HRP substrate was purchased from Merck KGaA (Merck Millipore, Burlington, MA, USA).

### 2.2. Cell Culture

SH-SY5Y cells (ATCC^®^ CRL-2266™; Manassas, VA, USA) were used as an in vitro neuronal cell model. The stock cultures were tested for mycoplasma contamination before cryopreservation. Cells were cultured in DMEM supplemented with 10% FBS, 1% penicillin–streptomycin, and 1% NEAA at 37 °C in a humidified atmosphere containing 5% CO_2_. The culture medium was replaced every 2–3 days, and cells were subcultured at approximately 80–90% confluence. Cells at passages 18–20 were used for all experiments [13].

### 2.3. Cell Viability Assay

Cell viability was determined by the MTT reduction assay. SH-SY5Y cells were dispensed into 96-well plates at 1 × 10^4^ cells/well and allowed to adhere overnight before treatment. To establish the oxidative stress condition, cells were exposed to increasing concentrations of H_2_O_2_ (0–800 μM) for 24 h. In our previous study using the same SH-SY5Y cell model, we observed a concentration-dependent decrease in cell viability following H_2_O_2_ exposure. Based on these findings, 200 μM H_2_O_2_ was selected for the present study as it induced a reproducible and moderate level of cellular injury while maintaining sufficient cell viability for the assessment of cytoprotective effects [13].

The cytotoxicity of KAE and NAC was first examined by exposing cells to a range of concentrations of each compound for 24 h. Concentrations that did not significantly affect basal cell viability were subsequently used for the protection experiments. For the oxidative stress model, cells were pretreated with KAE, NAC, or KNAC for 24 h. Following pretreatment, the culture medium was replaced with fresh medium containing H_2_O_2_ and the corresponding treatments, and the cells were maintained under these conditions for a further 24 h. Untreated cells served as the control group, whereas cells exposed to H_2_O_2_ alone represented the oxidative stress group.

At the end of the treatment period, MTT solution was added to each well to obtain a final concentration of 0.5 mg/mL. The plates were incubated at 37 °C for 1 h, after which the medium was removed, and the formazan crystals were solubilized in 100 μL DMSO. Absorbance was measured at 570 nm using a Synergy H1 Hybrid Multi-Mode Reader (BioTek Instruments, Winooski, VT, USA). Background absorbance was corrected before cell viability was calculated relative to the untreated cell control, which was defined as 100%. Cell viability (%) was calculated using the following equation:

Cell viability (%) = [(A_t_ − A_b_)/(A_c_ − A_b_)] × 100

where A_t_ is the absorbance of the treatment group, A_c_ is the absorbance of the untreated cell control, and A_b_ is the corresponding background absorbance. Cell viability was used to evaluate the cytotoxicity and protective effects of KAE, NAC, and KNAC [14].

To assess potential interference of KAE and NAC with the MTT assay, cell-free controls containing the corresponding concentrations of each compound were processed using the same experimental procedures as the cell-containing samples, except that no cells were added. Vehicle-treated cell-free samples served as the reference controls. Background-corrected absorbance values from the cell-free controls were expressed as percentage changes relative to the corresponding vehicle controls to evaluate compound-related changes in the MTT assay signal in the absence of cells.

### 2.4. Determination of Optimal Ratio and Combination Effects

Non-cytotoxic concentrations of KAE and NAC were first established using the cell viability assay described above. Five concentrations of each compound that did not significantly reduce cell viability were selected for subsequent combination experiments. Each compound was tested individually at the selected concentrations, and the two compounds were combined in a 5 × 5 concentration matrix, yielding 25 combination conditions. The single-agent treatments and the H_2_O_2_-treated vehicle control were included to evaluate the cytoprotective effects and potential interactions of KAE and NAC across the tested concentration ranges [14].

For the combination-response analysis, cell viability values were normalized to express the relative recovery of cell viability following H_2_O_2_-induced injury. The H_2_O_2_-treated vehicle control was used as the lower reference, whereas the untreated control was used as the upper reference. Normalized cell viability (%) was calculated as follows:

Normalized cell viability (%) = [(V_t_ − V_V_)/(V_C_ − V_V_)] × 100

where V_t_ represents the measured cell viability of each treatment group, V_V_ represents the cell viability of the H_2_O_2_-treated vehicle control, and V_C_ represents the cell viability of the untreated control. The resulting values represent the relative recovery of cell viability compared with the loss induced by H_2_O_2_, rather than absolute cell viability.

The interaction between KAE and NAC was evaluated using SynergyFinder (version R-3.10.3). The combination-response data were analyzed using all available reference models, including Bliss independence, Loewe additivity, highest single agent (HSA), and zero interaction potency (ZIP) models. Synergy scores were calculated for each model, and the corresponding synergy landscapes were visualized as two-dimensional contour plots. Because these models are based on different assumptions, the results were interpreted separately for each model rather than assuming a consistent synergistic interaction across all models.

Combination effects were further evaluated using the Chou–Talalay CI method:

CI = (D_1_/Dx_1_) + (D_2_/Dx_2_)

where D_1_ and D_2_ represent the concentrations of KAE and NAC, respectively, used in combination to produce a defined level of effect, whereas Dx_1_ and Dx_2_ represent the concentrations of KAE and NAC, respectively, required individually to produce the same effect. CI values were interpreted as follows: CI < 1 indicates synergism, CI = 1 indicates an additive interaction, and CI > 1 indicates antagonism [14].

The KAE-NAC combination selected for subsequent mechanistic experiments was determined based on the cytoprotective effects observed across the tested concentration combinations and the results of the interaction analyses, including CI values and the SynergyFinder reference models. The selected combination was designated as KNAC and used to investigate antioxidant responses associated with SIRT3/FOXO3a/SOD2-mediated mitochondrial defense and the GSH-dependent cellular redox system.

### 2.5. Measurement of Intracellular Oxidant-Sensitive Fluorescence

Intracellular oxidant-sensitive fluorescence was assessed using CM-H_2_DCFDA as a fluorescent indicator of cellular oxidative status. SH-SY5Y cells were seeded in 96-well plates and allowed to attach overnight. Cells were then treated with KAE, NAC, or KNAC according to the experimental conditions described above. After the pretreatment period, the cells were incubated with 10 μM CM-H_2_DCFDA at 37 °C for 30 min in the dark. The cells were subsequently washed with PBS to remove unincorporated probe.

Oxidative stress was induced by adding H_2_O_2_ to the corresponding treatment groups, followed by incubation for the designated experimental period. Fluorescence was measured using a Synergy H1 Hybrid Multi-Mode Reader at an excitation wavelength of 488 nm and an emission wavelength of 520 nm. The fluorescence intensity of each treatment group was normalized to that of the untreated control and expressed as relative CM-H_2_DCFDA fluorescence [15].

### 2.6. Measurement of Lipid Peroxidation

Lipid peroxidation was evaluated by measuring MDA using a TBARS-based assay. Briefly, cell samples were combined with SDS, TBA, and acetic acid and heated at 95 °C for 10 min. The reaction mixtures were subsequently allowed to cool to 25 °C before extraction of the reaction products with n-butanol:pyridine (15:1, *v*/*v*).

The samples were centrifuged at 4000× *g* for 10 min, and the upper organic phase was transferred for spectrophotometric analysis. Absorbance was measured at 532 nm using a Synergy H1 Hybrid Multi-Mode Reader. MDA concentrations were determined from a standard curve generated with TMP at concentrations ranging from 0 to 15 μM and expressed as MDA equivalents [16].

### 2.7. Measurement of Antioxidant Enzyme Activities

CAT activity was determined based on the enzymatic decomposition of H_2_O_2_. An aliquot of the cell homogenate (10 μL) was added to a reaction mixture containing 50 μL of 30 mM H_2_O_2_ prepared in 50 mM phosphate buffer (pH 7.0), followed by the addition of 150 μL of 5 mM KMnO_4_ and 25 μL of 4 M H_2_SO_4_. The absorbance of the reaction mixture was measured at 490 nm. A CAT standard curve prepared over the range of 10–100 units/mL was used for activity quantification. CAT activity was expressed as units/mg protein [16].

SOD activity was determined using a xanthine–xanthine oxidase system with cytochrome c as the indicator substrate. The reaction mixture was prepared from 0.5 mM xanthine (pH 7.4), 0.2 M potassium phosphate buffer (pH 7.8), 0.01 M EDTA, and cytochrome c at a volume ratio of 50:25:1:1. Cell homogenate (20 μL) was added to 200 μL of the reaction mixture, followed by 20 μL of xanthine oxidase (0.90 mU/mL). Absorbance was recorded at 415 nm. SOD standards ranging from 0 to 25 units/mL were used to generate the calibration curve, and enzyme activity was expressed as units/mg protein [16].

GSH-Px activity was assessed using a reaction system containing DTT, sodium phosphate, sodium azide, GSH, and H_2_O_2_. Briefly, 20 μL of cell homogenate was combined with the reaction mixture and incubated at 25 °C for 10 min. DTNB was then added to the reaction, and the absorbance was measured at 412 nm. A GSH-Px standard curve prepared using concentrations ranging from 1 to 5 units/mL was used to quantify enzyme activity. GSH-Px activity was normalized to protein content and expressed as units/mg protein [16].

### 2.8. Measurement of Intracellular GSH and GSSG Levels

Intracellular GSH and GSSG were quantified using the Glutathione Assay Kit according to the manufacturer’s protocol. Briefly, cells from each experimental group were collected and prepared as cell lysates, followed by deproteinization of the samples before glutathione analysis. The resulting supernatants were appropriately diluted with assay buffer to obtain values within the linear range of the standard curve.

For total glutathione determination, the samples and glutathione standards were combined with the assay reagents and incubated to allow the enzymatic recycling reaction to proceed. Absorbance was measured at 405 nm using a Synergy H1 Hybrid Multi-Mode Reader. GSSG was analyzed separately using the GSSG-specific procedure, in which GSH was masked prior to the assay to minimize interference from reduced glutathione. GSH levels were subsequently determined from the total glutathione and GSSG measurements. Standard curves were generated from the corresponding glutathione standards, and sample concentrations were calculated after accounting for dilution factors.

The GSH/GSSG ratio was calculated for each treatment group as an index of intracellular redox balance. Glutathione levels were normalized to the protein content of the corresponding cell lysates and expressed as nmol/mg protein.

### 2.9. Western Blot Analysis

Protein expression was examined by Western blotting following the respective treatment protocols. SH-SY5Y cells were collected and lysed in neuronal protein extraction reagent (N-PER™) supplemented with a protease inhibitor cocktail. The lysates were centrifuged at 10,000× *g* for 10 min at 4 °C, and the clarified supernatants were collected for protein quantification. Equal amounts of protein (60 μg per sample) were mixed with loading buffer and heated at 95 °C for 5 min before electrophoretic separation by SDS-PAGE.

Following electrophoresis, proteins were transferred onto polyvinylidene fluoride (PVDF) membranes. The membranes were blocked with 5% skim milk in TBS-T and subsequently incubated overnight at 4 °C with primary antibodies against SIRT3, FOXO3a, SOD2, and β-actin (all at 1:1000 dilution). After washing with TBS-T, the membranes were incubated with HRP-linked anti-rabbit IgG secondary antibody (1:2000 dilution) for 1 h at 25 °C. Protein bands were detected using Immobilon^®^ Forte Western HRP substrate and visualized with a ChemiDoc™ MP Imaging System (Bio-Rad Laboratories, Hercules, CA, USA).

Band intensity was quantified using Image Lab software (version 6.0.0; Bio-Rad Laboratories) and normalized to β-actin. The expression levels of SIRT3, FOXO3a, and SOD2 were presented relative to the untreated control group [17].

### 2.10. Molecular Docking Simulation

The crystal structure of human SIRT3 (PDB ID: 3GLS) was obtained from the Protein Data Bank, based on the structure reported by Jin et al. [18]. The structure was determined by X-ray diffraction at a resolution of 2.70 Å. Kaempferol (KAE; CID: 5280863) and N-acetyl-L-cysteine (NAC; CID: 12035) were selected as ligands for the docking analysis. Before docking, the protein and ligand structures were prepared by adding hydrogen atoms and removing unnecessary molecular components. The receptor was prepared by removing co-crystallized water molecules and non-essential heteroatoms, adding polar hydrogens, assigning partial charges, and converting the structure to PDBQT format.

Molecular docking was performed using AutoDock Vina version 1.2.0 [19]. A total of 150 independent docking runs were performed, with a maximum of 25,000,000 energy evaluations and a population size of 300. The grid box was centered at X = 26.037, Y = 46.249, and Z = −1.103 Å, with dimensions of 70 × 70 × 70 grid points and a grid spacing of 0.375 Å. The receptor was treated as rigid, without explicitly defined flexible residues. The resulting poses were ranked according to their predicted binding energies, expressed in kcal/mol, with more negative values indicating more favorable predicted binding. The docked complexes were analyzed using BIOVIA Discovery Studio Visualizer (Dassault Systèmes, San Diego, CA, USA). Three-dimensional binding poses and two-dimensional interaction diagrams were generated to identify the amino acid residues involved in the predicted ligand–SIRT3 interactions. The interactions were classified as conventional hydrogen bonds, π–π interactions, π–alkyl interactions, or π–sigma interactions, and the interacting residues were recorded for comparison between KAE and NAC.

### 2.11. Statistical Analysis

Results are expressed as the mean ± SEM from independent experiments. Statistical analyses were conducted using IBM SPSS Statistics for Mac, version 28.0 (IBM Corp., Armonk, NY, USA). Differences among multiple treatment groups were evaluated by one-way ANOVA followed by Tukey’s multiple-comparison test, whereas comparisons between two groups were performed using an independent-samples Student’s *t*-test. A two-sided *p*-value < 0.05 was considered statistically significant.

## 3. Results

### 3.1. Cytotoxicity of KAE and NAC

**Figure 1:**
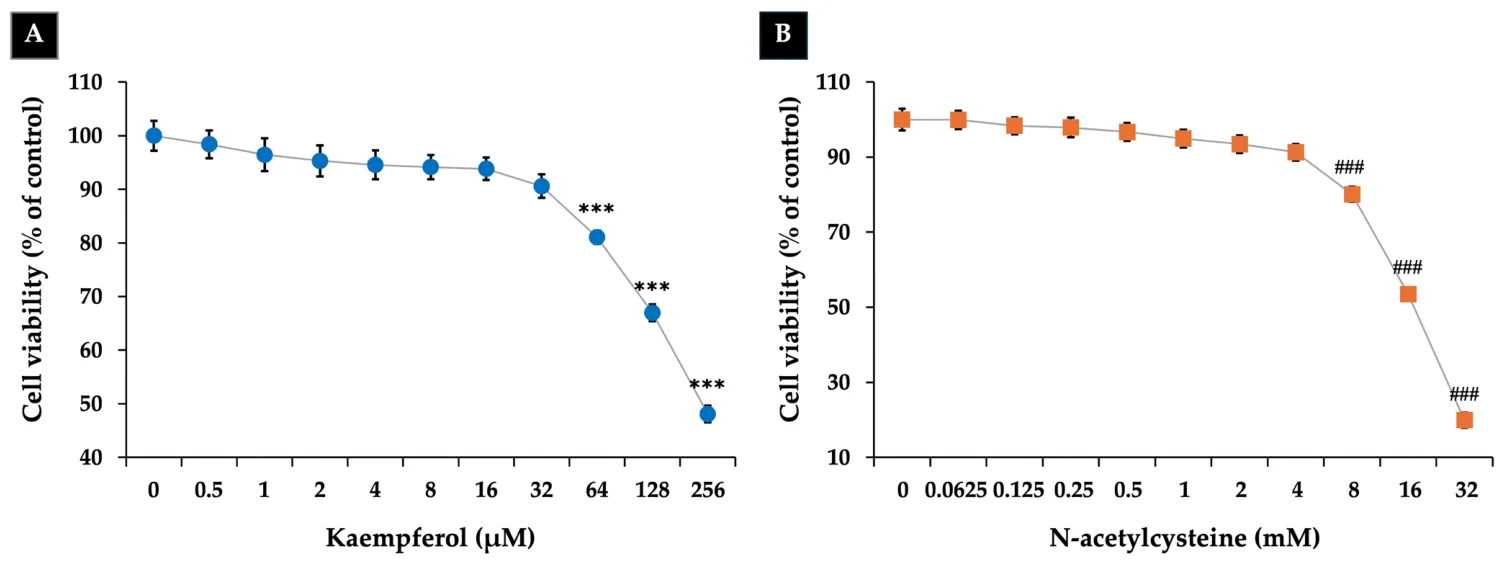
Cytotoxicity of kaempferol (KAE) and N-acetylcysteine (NAC) in SH-SY5Y cells. Cell viability was assessed after treatment with KAE or NAC at the indicated concentrations for 24 h using the MTT assay. Data are presented as mean ± SEM from three independent experiments. ***, *p* < 0.001 compared with the 0 μM KAE control; ###, *p* < 0.001 compared with the 0 mM NAC control.

The cytotoxicity of KAE and NAC was evaluated in SH-SY5Y cells over concentration ranges of 0–256 μM and 0–32 mM, respectively (Figure 1A,B). KAE did not significantly affect cell viability at concentrations up to 32 μM, whereas concentrations of 64, 128, and 256 μM significantly reduced cell viability compared with the untreated control (all *p* < 0.001). Thus, 32 μM was selected as the highest non-cytotoxic concentration of KAE, maintaining cell viability above 90%.

Similarly, NAC did not significantly reduce cell viability at concentrations up to 4 mM. In contrast, NAC at 8, 16, and 32 mM significantly decreased cell viability compared with the untreated control (all *p* < 0.001). Therefore, 4 mM was selected as the highest non-cytotoxic concentration of NAC for subsequent experiments.

To assess potential interference with the MTT assay, cell-free controls were evaluated over the concentration ranges used in the subsequent combination experiments (KAE, 2–32 μM; NAC, 0.25–4 mM) using the same experimental procedures as the cell-containing samples, except that no cells were added. The changes in cell-free MTT signals relative to the corresponding vehicle controls were relatively small across these concentration ranges (Supplementary Figure S1).

### 3.2. Combination Effects of KAE and NAC

**Figure 2:**
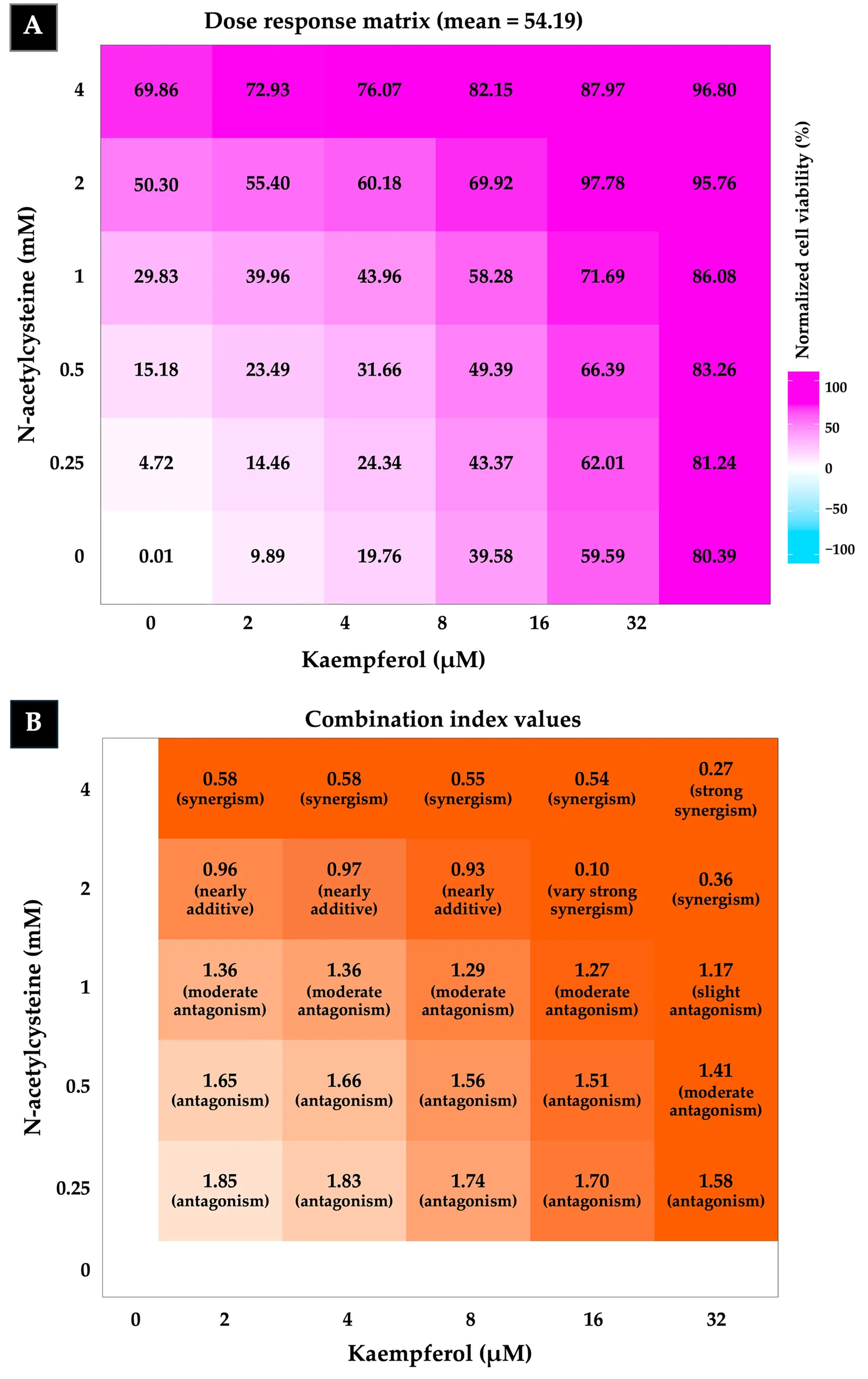
Combination effects of kaempferol (KAE) and N-acetylcysteine (NAC) in H_2_O_2_-treated SH-SY5Y cells. (**A**) Dose–response matrix showing the normalized cell viability values, expressed as relative recovery of cell viability following H_2_O_2_-induced injury, across the tested KAE–NAC combinations. (**B**) Combination index (CI) values obtained from the KAE–NAC combinations, indicating synergistic (CI < 1), additive (CI = 1), or antagonistic (CI > 1) interactions.

Based on the cytotoxicity results, five non-cytotoxic concentrations of KAE (2, 4, 8, 16, and 32 μM) and NAC (0.25, 0.5, 1, 2, and 4 mM) were selected for combination experiments. The compounds were tested using a 5 × 5 concentration matrix, resulting in 25 KAE–NAC combinations (Figure 2A). The normalized cell viability values across the combination matrix ranged from 14.46% to 97.78%, with a mean of 54.19%, indicating varying degrees of recovery from H_2_O_2_-induced cellular injury. The greatest recovery was observed with 16 μM KAE combined with 2 mM NAC.

The interaction between KAE and NAC was further assessed using CI analysis (Figure 2B). Antagonistic interactions were predominantly observed at lower NAC concentrations (0.25–1 mM), with CI values ranging from 1.85 to 1.36. In contrast, combinations containing 2–4 mM NAC showed additive to synergistic interactions, with CI values ranging from 0.10 to 0.97. The strongest synergistic interaction was observed with 16 μM KAE and 2 mM NAC (CI = 0.10), corresponding to a KAE:NAC molar ratio of 1:125.

### 3.3. Synergy Analysis of KAE and NAC

**Figure 3:**
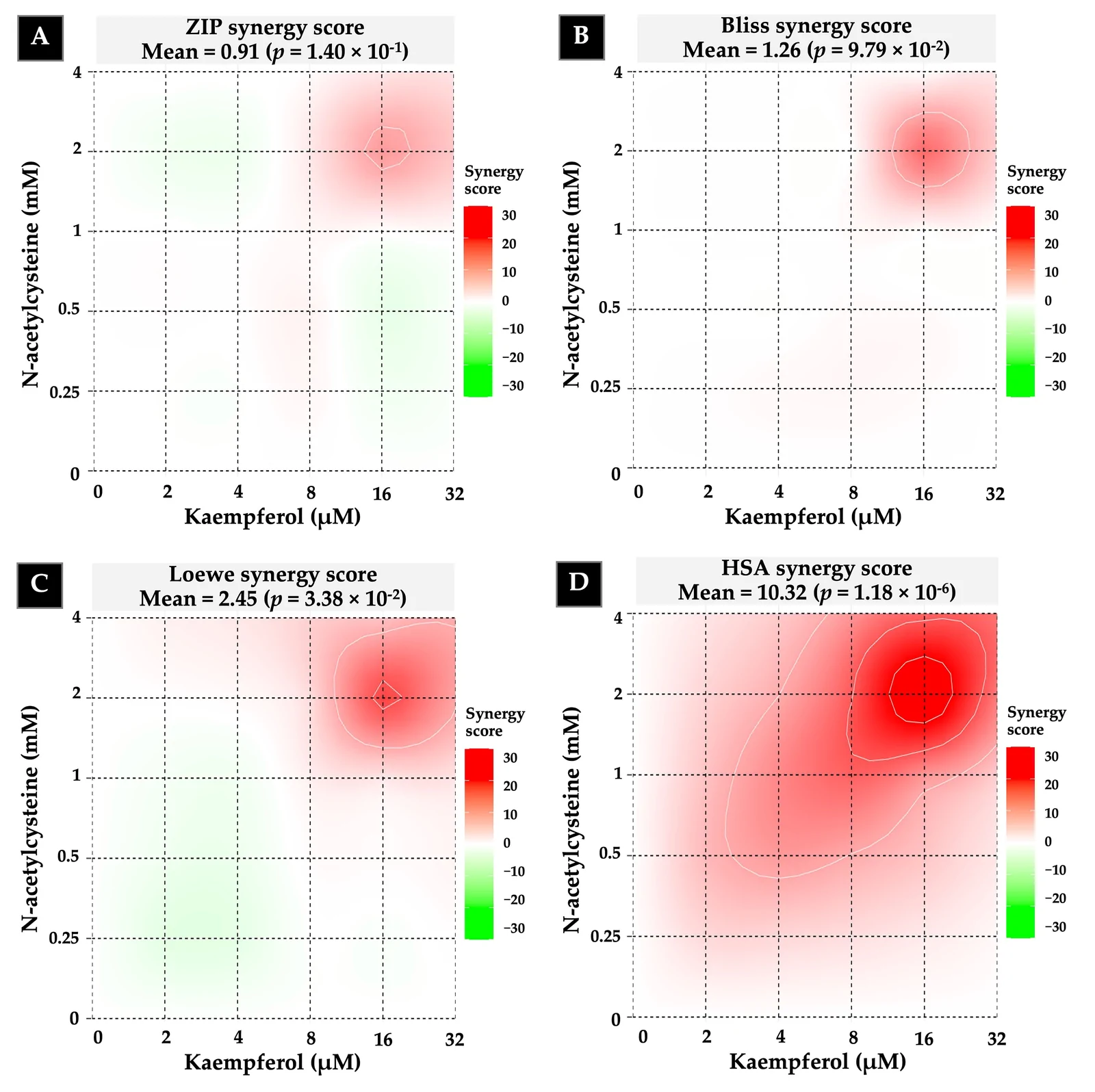
Synergistic effects of kaempferol (KAE) and N-acetylcysteine (NAC) in SH-SY5Y cells. (**A**) Zero interaction potency (ZIP) synergy score, (**B**) bliss synergy score, (**C**) Loewe synergy score, and (**D**) highest single agent (HSA) synergy score for the combination of KAE and NAC. Red and green areas indicate synergistic and antagonistic effects, respectively. Data are presented as mean synergy scores with the corresponding *p*-values.

The synergistic effects of the 25 KAE–NAC combinations were further evaluated using four reference models in SynergyFinder (Figure 3). Positive mean synergy scores were observed across all models, with mean scores of 0.91 for ZIP, 1.26 for Bliss, 2.45 for Loewe, and 10.32 for HSA. The Loewe and HSA models showed statistically significant synergy scores (*p* = 0.0338 and *p* = 1.18 × 10^−6^, respectively), whereas the ZIP and Bliss models did not reach statistical significance (*p* = 0.140 and 0.0979, respectively). These findings indicate that the evidence for synergy depended on the reference model, with statistically significant results obtained using the Loewe and HSA models but not the Bliss and ZIP models. Therefore, the interaction between KAE and NAC should be interpreted as model-dependent rather than consistently synergistic across all four models.

### 3.4. Protective Effects of KNAC on H_2_O_2_-Induced Cytotoxicity

**Figure 4:**
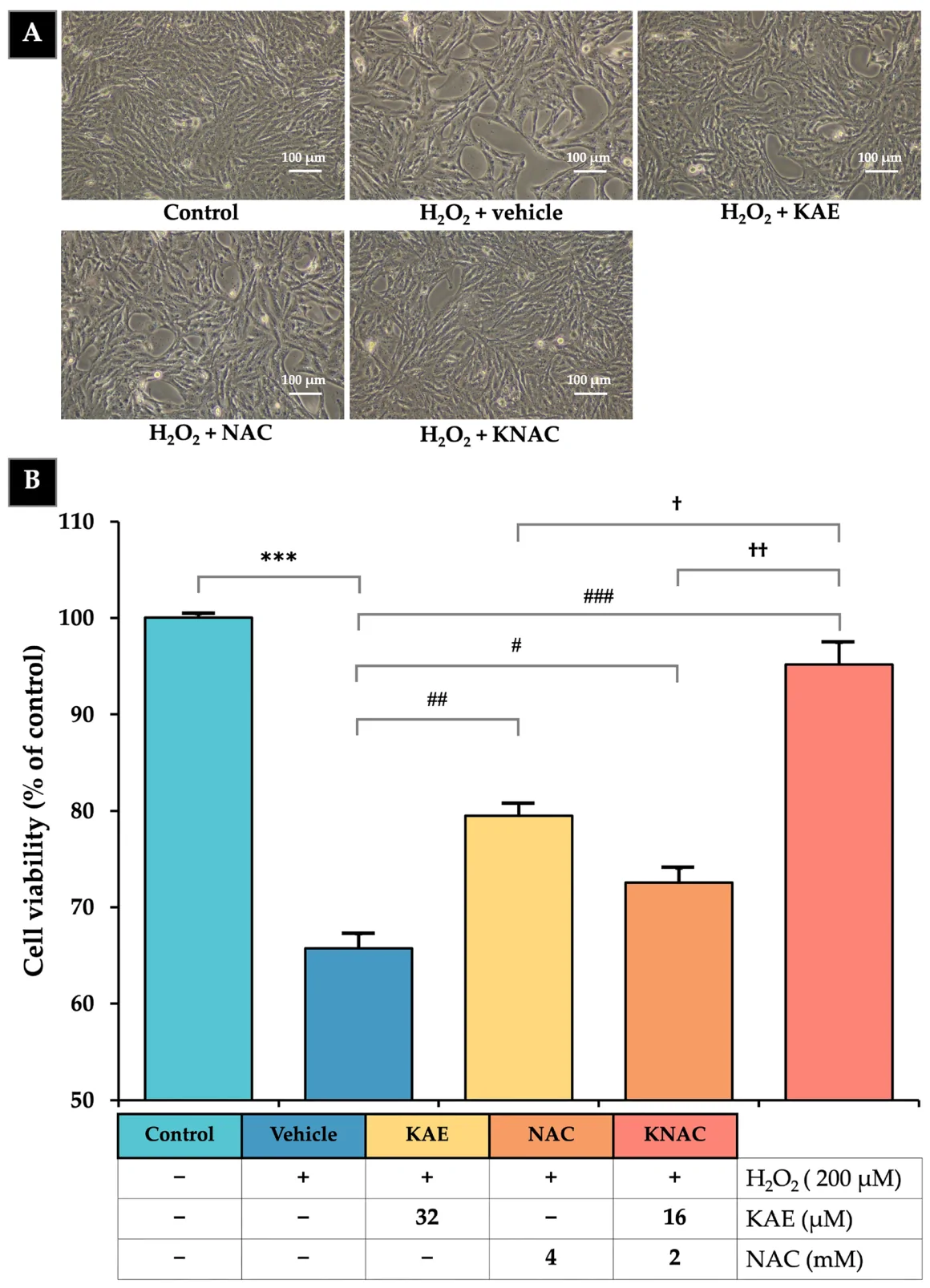
Protective effects of kaempferol (KAE), N-acetylcysteine (NAC), and their combination (KNAC) against H_2_O_2_-induced cytotoxicity in SH-SY5Y cells. (**A**) Representative images of SH-SY5Y cells observed under a phase-contrast microscope at 10× magnification. (**B**) Cell viability following treatment with KAE, NAC, or their combination (KNAC) in the presence of H_2_O_2_. Data are presented as mean ± SEM from three independent experiments. ***, *p* < 0.001 compared with the control group; #, ##, and ###, *p* < 0.05, *p* < 0.01, and *p* < 0.001, respectively, compared with the H_2_O_2_ + vehicle group; † and ††, *p* < 0.05 and *p* < 0.01, respectively, compared with the KNAC group.

To evaluate the protective effects of KAE, NAC, or KNAC against H_2_O_2_-induced cytotoxicity, cell morphology and viability were assessed in SH-SY5Y cells following H_2_O_2_ exposure. Representative phase-contrast images of SH-SY5Y cells are shown at 10× magnification (Figure 4A). Exposure to H_2_O_2_ markedly reduced cell viability compared with the control group (*p* < 0.001; Figure 4B). Treatment with KAE, NAC, or their combination significantly improved cell viability compared with the H_2_O_2_ + vehicle group, with *p*-values of <0.01, <0.05, and <0.001, respectively. Notably, the KNAC treatment resulted in significantly higher cell viability than KAE alone (*p* < 0.05) and NAC alone (*p* < 0.01).

### 3.5. Effects of KNAC on Oxidative Stress

**Figure 5:**
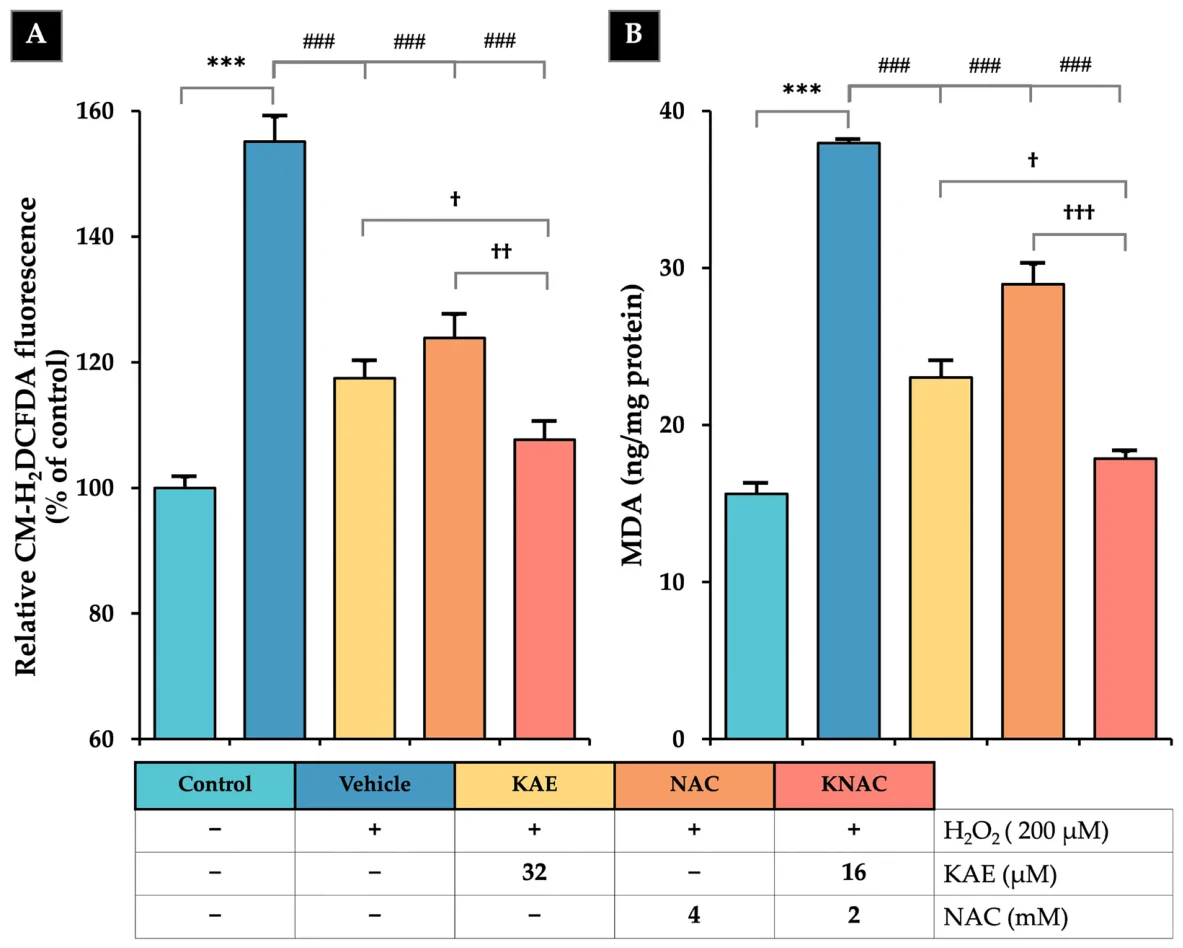
Effects of kaempferol (KAE), N-acetylcysteine (NAC), and their combination (KNAC) on oxidative stress in H_2_O_2_-treated SH-SY5Y cells. (**A**) Relative CM-H_2_DCFDA fluorescence. (**B**) Malondialdehyde (MDA) levels. Data are presented as mean ± SEM from three independent experiments. ***, *p* < 0.001 compared with the control group; ###, *p* < 0.001 compared with the H_2_O_2_ + vehicle group; †, ††, and †††, *p* < 0.05, *p* < 0.01, and *p* < 0.001, respectively, compared with the KNAC group.

Following the improvement in cell viability observed with KAE, NAC, and KNAC, CM-H_2_DCFDA fluorescence and MDA levels were assessed to further characterize their effects on H_2_O_2_-induced oxidative stress (Figure 5). H_2_O_2_ + vehicle significantly increased CM-H_2_DCFDA fluorescence compared with the control group (*p* < 0.001; Figure 5A). Treatment with KAE, NAC, and KNAC significantly reduced CM-H_2_DCFDA fluorescence compared with the H_2_O_2_ + vehicle group (all *p* < 0.001). Notably, the KNAC treatment produced a greater reduction in CM-H_2_DCFDA fluorescence than KAE alone (*p* < 0.05) and NAC alone (*p* < 0.01).

Similarly, MDA levels were significantly higher in the H_2_O_2_ + vehicle group than in the control group (*p* < 0.001; Figure 5B). Treatment with KAE, NAC, and KNAC significantly reduced MDA levels compared with the H_2_O_2_ + vehicle group (all *p* < 0.001). Notably, KNAC reduced MDA levels more effectively than KAE alone (*p* < 0.05) and NAC alone (*p* < 0.01).

### 3.6. Effects of KNAC on Antioxidant Enzyme Activities

**Figure 6:**
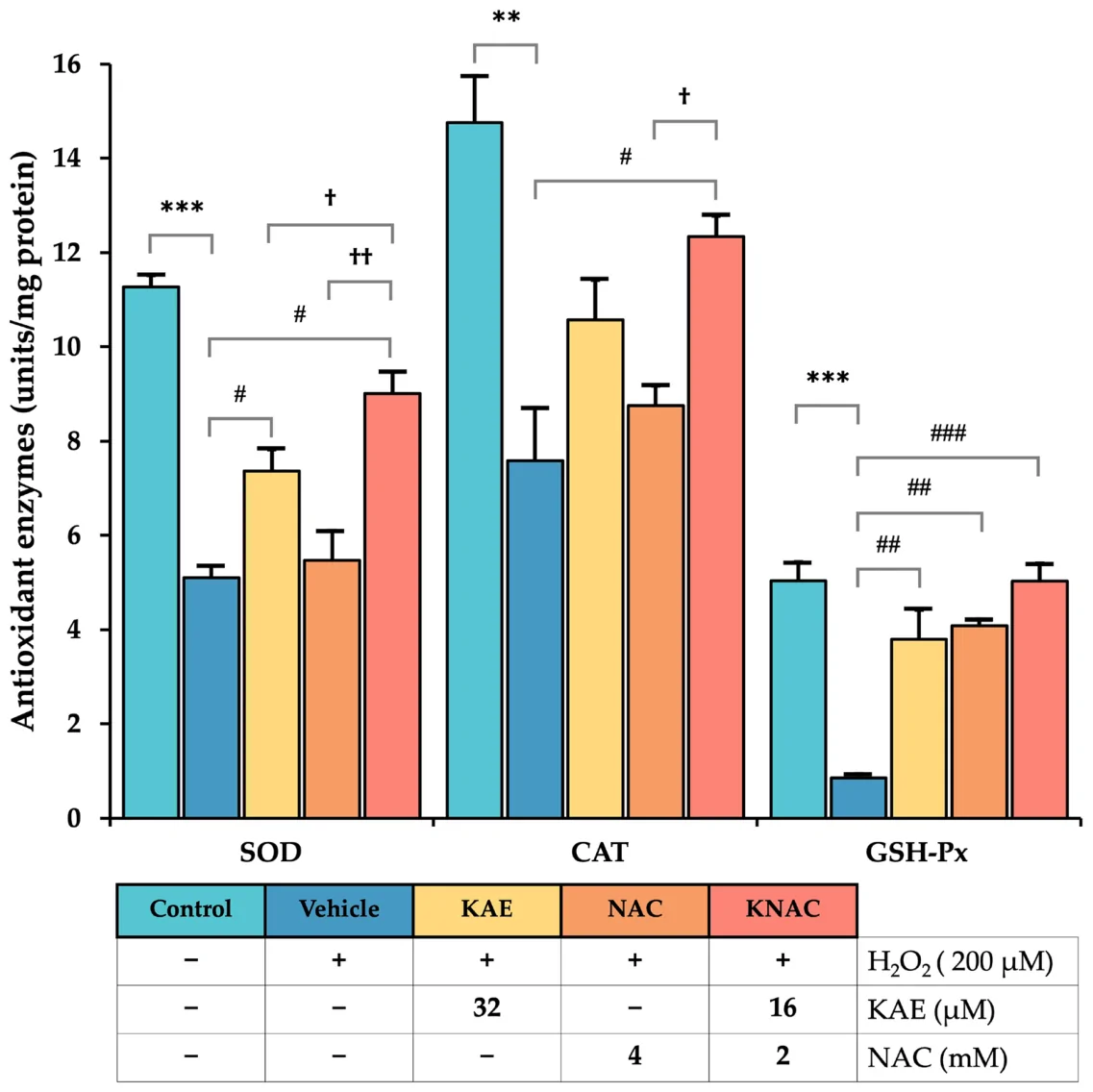
Effects of kaempferol (KAE), N-acetylcysteine (NAC), and their combination (KNAC) on antioxidant enzyme activities in H_2_O_2_-treated SH-SY5Y cells. The activities of superoxide dismutase (SOD), catalase (CAT), and glutathione peroxidase (GSH-Px) were measured. Data are presented as mean ± SEM from three independent experiments. ** and ***, *p* < 0.01 and *p* < 0.001, respectively, compared with the control group; #, ##, and ###, *p* < 0.05, *p* < 0.01, and *p* < 0.001, respectively, compared with the H_2_O_2_ + vehicle group; † and ††, *p* < 0.05 and *p* < 0.01, respectively, compared with the KNAC group.

To further evaluate the effects of KAE, NAC, and KNAC on the antioxidant defense system following H_2_O_2_ exposure, the activities of SOD, CAT, and GSH-Px were assessed (Figure 6). H_2_O_2_ exposure significantly altered antioxidant enzyme activities compared with the control group, while treatment with KAE, NAC, or KNAC showed differential effects on the individual antioxidant enzymes.

SOD activity was significantly lower in the H_2_O_2_ + vehicle group than in the control group (*p* < 0.001). Treatment with KAE and KNAC significantly increased SOD activity compared with the H_2_O_2_ + vehicle group (both *p* < 0.05), whereas NAC alone did not produce a significant change. Notably, SOD activity in the KNAC group was significantly higher than that in the KAE group (*p* < 0.05) and NAC group (*p* < 0.01).

Similarly, CAT activity was significantly reduced by H_2_O_2_ exposure compared with the control group (*p* < 0.01). Among the treatments, only KNAC significantly increased CAT activity compared with the H_2_O_2_ + vehicle group (*p* < 0.05). CAT activity in the KNAC group was also significantly higher than that in the NAC group (*p* < 0.05).

For GSH-Px activity, the H_2_O_2_ + vehicle group showed a significant decrease compared with the control group (*p* < 0.001). KAE, NAC, and KNAC significantly increased GSH-Px activity compared with the H_2_O_2_ + vehicle group, with *p*-values of <0.01, <0.01, and <0.001, respectively. Although GSH-Px activity was higher in the KNAC group than in the groups treated with KAE or NAC alone, these differences did not reach statistical significance.

### 3.7. Effects of KNAC on SIRT3 Expression

**Figure 7:**
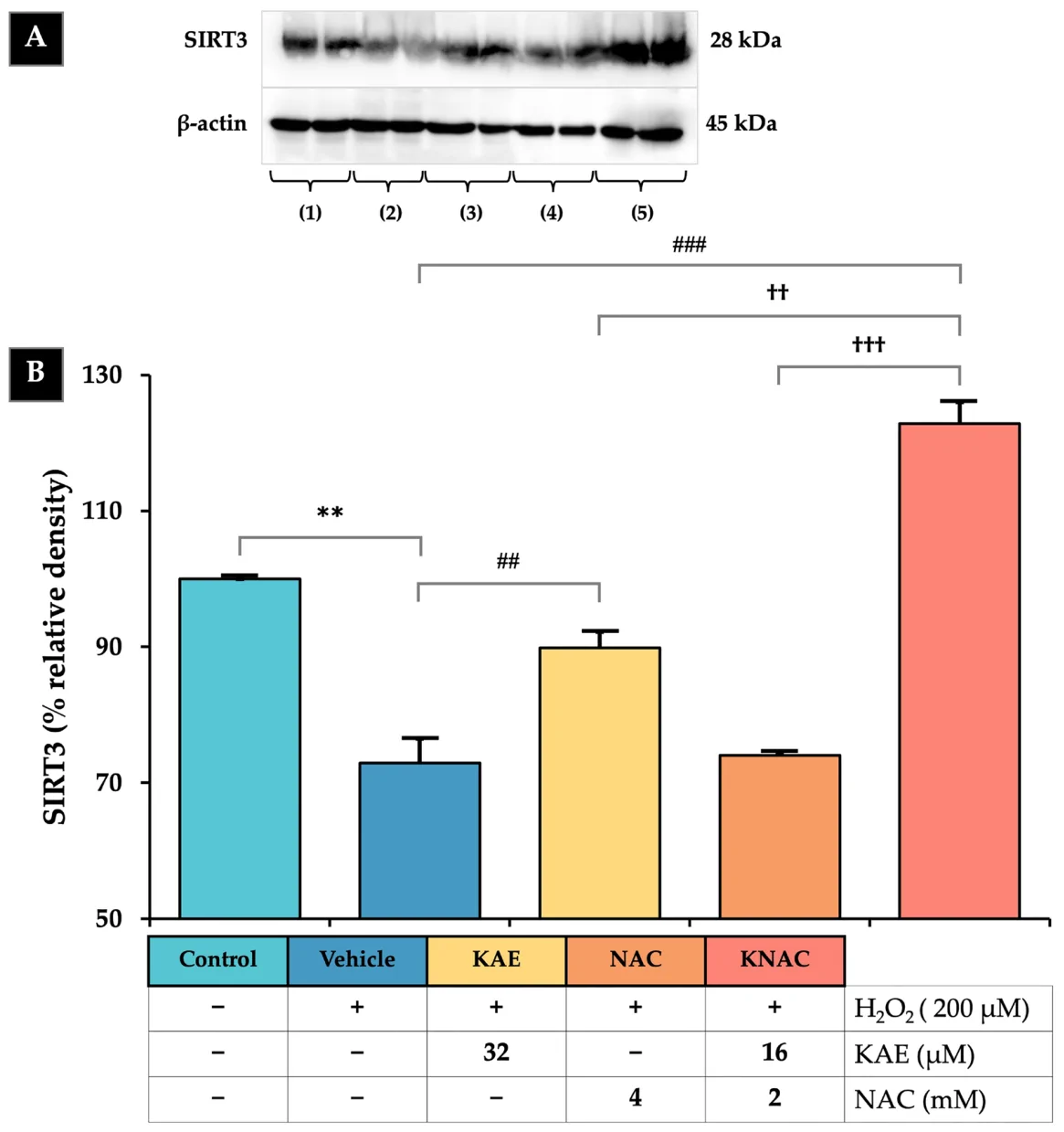
Effects of kaempferol (KAE), N-acetylcysteine (NAC), and their combination (KNAC) on sirtuin 3 (SIRT3) expression in H_2_O_2_-treated SH-SY5Y cells. (**A**) Representative Western blot images of SIRT3 and β-actin. (**B**) Relative density of SIRT3 normalized to β-actin. Data are presented as mean ± SEM from three independent experiments. **, *p* < 0.01 compared with the control group; ## and ###, *p* < 0.01 and *p* < 0.001, respectively, compared with the H_2_O_2_ + vehicle group; †† and †††, *p* < 0.01 and *p* < 0.001, respectively, compared with the KNAC group.

To further investigate the potential mechanism underlying the antioxidant effects of KAE, NAC, and KNAC, SIRT3 protein expression was evaluated following H_2_O_2_ exposure (Figure 7). SIRT3 expression was significantly reduced in the H_2_O_2_ + vehicle group compared with the control group (*p* < 0.01). Treatment with KAE and KNAC significantly increased SIRT3 expression compared with the H_2_O_2_ + vehicle group (*p* < 0.01 and *p* < 0.001, respectively), whereas NAC alone did not produce a significant change. Notably, SIRT3 expression in the KNAC group was significantly higher than that in the KAE group (*p* < 0.01) and NAC group (*p* < 0.001).

### 3.8. Effects of KNAC on FOXO3a and SOD2 Expression

**Figure 8:**
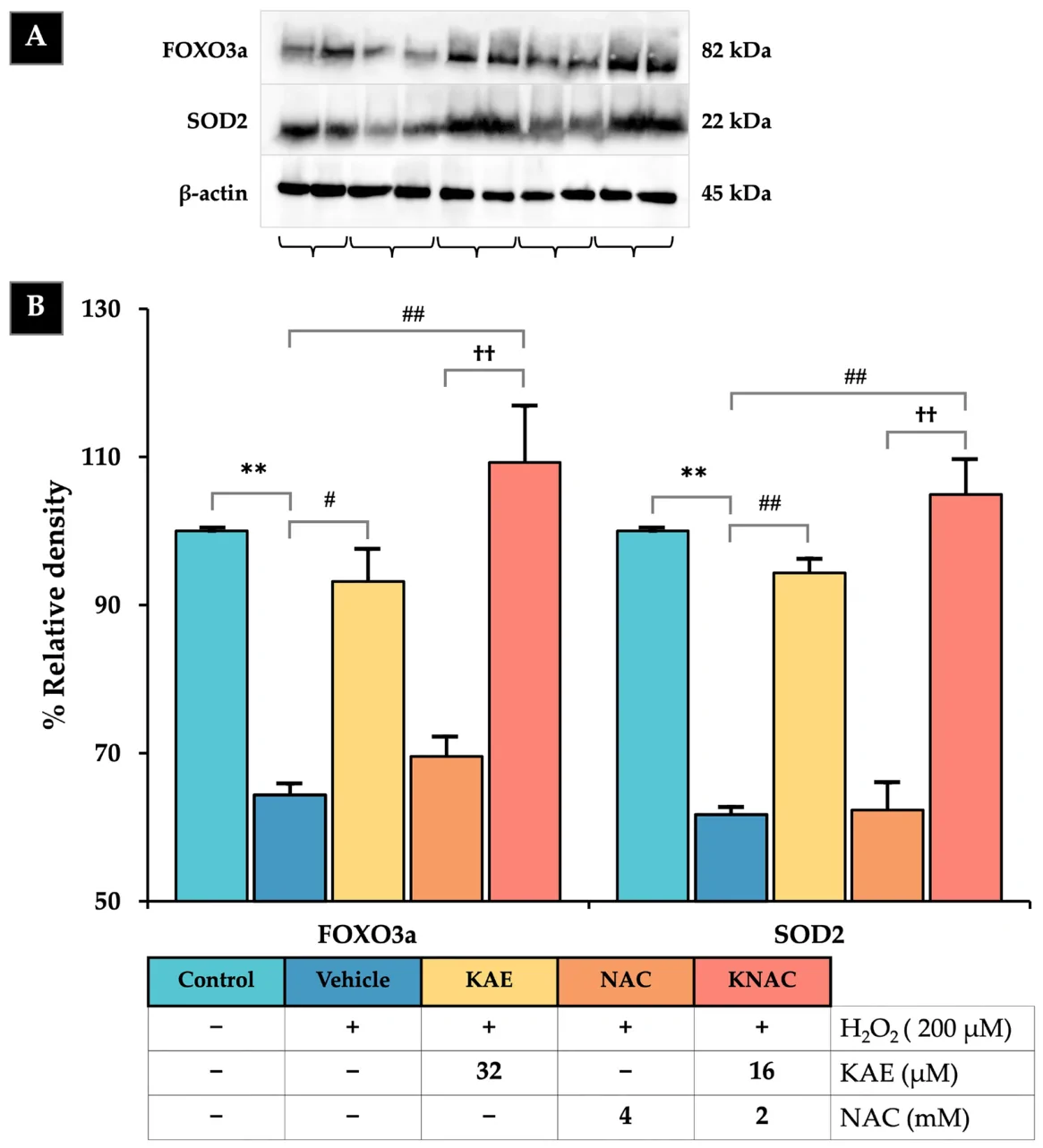
Effects of kaempferol (KAE), N-acetylcysteine (NAC), and their combination (KNAC) on Forkhead box O3 (FOXO3a) and superoxide dismutase 2 (SOD2) expression in H_2_O_2_-treated SH-SY5Y cells. (**A**) Representative Western blot images of FOXO3a, SOD2, and β-actin. (**B**) Relative density of FOXO3a and SOD2 normalized to β-actin. Data are presented as mean ± SEM from three independent experiments. **, *p* < 0.01 compared with the control group; # and ##, *p* < 0.05 and *p* < 0.01, respectively, compared with the H_2_O_2_ + vehicle group; ††, *p* < 0.01 compared with the KNAC group.

To further examine the downstream antioxidant pathway associated with SIRT3, the protein expression levels of FOXO3a and SOD2 were evaluated following H_2_O_2_ exposure (Figure 8). FOXO3a expression was significantly reduced in the H_2_O_2_ + vehicle group compared with the control group (*p* < 0.01). Treatment with KAE and KNAC significantly increased FOXO3a expression compared with the H_2_O_2_ + vehicle group (*p* < 0.05 and *p* < 0.01, respectively), whereas NAC alone did not produce a significant change. Notably, FOXO3a expression in the KNAC group was significantly higher than that in the NAC group (*p* < 0.01).

Similarly, SOD2 expression was significantly reduced in the H_2_O_2_ + vehicle group compared with the control group (*p* < 0.01). KAE and KNAC significantly increased SOD2 expression compared with the H_2_O_2_ + vehicle group (both *p* < 0.01), whereas NAC alone did not produce a significant change. Notably, SOD2 expression in the KNAC group was significantly higher than that in the NAC group (*p* < 0.01).

### 3.9. Effects of KNAC on Glutathione Redox Status

To further evaluate the effects of KAE, NAC, and KNAC on cellular redox homeostasis, total glutathione, GSH, GSSG, and the GSH/GSSG ratio were assessed in H_2_O_2_-exposed SH-SY5Y cells (Table 1).

Total glutathione levels were significantly lower in the H_2_O_2_ + vehicle group than in the control group (*p* < 0.01). Treatment with NAC and KNAC significantly increased total glutathione levels compared with the H_2_O_2_ + vehicle group (*p* < 0.05 and *p* < 0.01, respectively), whereas KAE alone did not produce a significant change. Notably, total glutathione levels were significantly higher in the KNAC group than in the KAE group (*p* < 0.05).

Similarly, GSH levels were significantly reduced in the H_2_O_2_ + vehicle group compared with the control group (*p* < 0.001). NAC and KNAC significantly increased GSH levels compared with the H_2_O_2_ + vehicle group (*p* < 0.01 and *p* < 0.001, respectively), whereas KAE alone did not produce a significant change. Notably, the KNAC group showed significantly higher GSH levels than the KAE group (*p* < 0.01).

In contrast, GSSG levels were significantly increased in the H_2_O_2_ + vehicle group compared with the control group (*p* < 0.001). Treatment with KAE, NAC, and KNAC significantly reduced GSSG levels compared with the H_2_O_2_ + vehicle group (*p* < 0.001 for all comparisons). Notably, GSSG levels in the KNAC group were significantly lower than those in the KAE group (*p* < 0.001).

The GSH/GSSG ratio was markedly reduced in the H_2_O_2_ + vehicle group compared with the control group (*p* < 0.001). Treatment with KAE, NAC, and KNAC significantly increased the GSH/GSSG ratio compared with the H_2_O_2_ + vehicle group, with *p*-values of <0.05, <0.001, and <0.001, respectively. Importantly, the GSH/GSSG ratio was significantly higher in the KNAC group than in both the KAE and NAC groups (*p* < 0.001 and *p* < 0.05, respectively).

### 3.10. Molecular Interaction of Kaempferol and NAC with SIRT3

**Figure 9:**
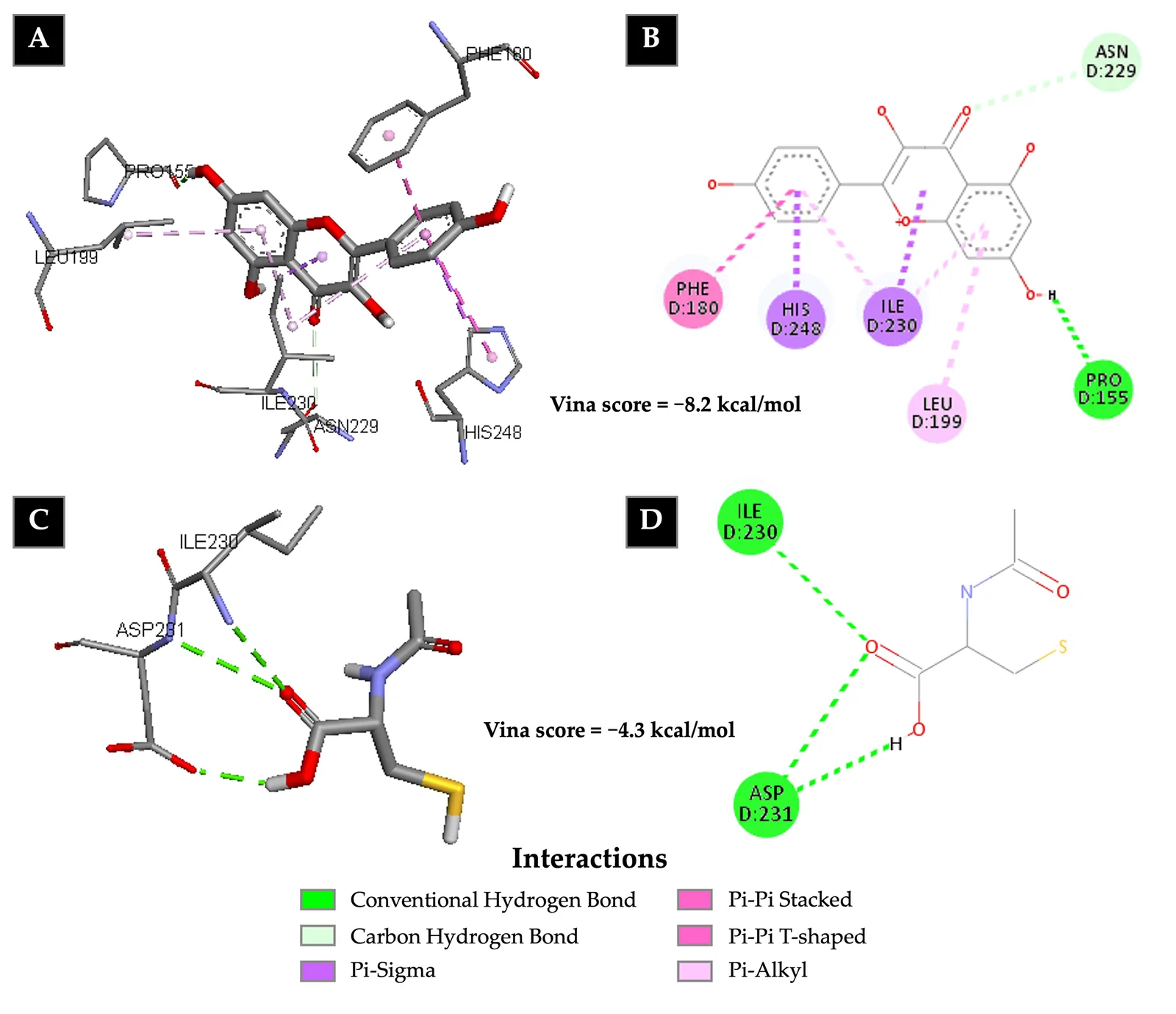
Molecular docking analysis of kaempferol (KAE) and N-acetylcysteine (NAC) with human sirtuin 3 (SIRT3) (PDB ID: 3GLS). (**A**) Three-dimensional representation of the SIRT3–KAE complex. (**B**) Two-dimensional interaction diagram of the SIRT3–KAE complex. (**C**) Three-dimensional representation of the SIRT3–NAC complex. (**D**) Two-dimensional interaction diagram of the SIRT3–NAC complex.

To further investigate the potential interaction of kaempferol and NAC with SIRT3, molecular docking analysis was performed, and the predicted interactions are summarized in Table 2 and Figure 9. Kaempferol showed a Vina score of −8.2 kcal/mol and was predicted to fit within the SIRT3 binding pocket. Several SIRT3 residues, including Pro155, Phe180, Leu199, Asn229, Ile230, and His248, were involved in non-covalent interactions with kaempferol. Among these interactions, aromatic and hydrophobic contacts involving Phe180, Leu199, Ile230, and His248, together with hydrogen-bond interactions involving Pro155 and Asn229, may contribute to stabilization of kaempferol within the binding pocket.

In comparison, NAC showed hydrogen-bond interactions with Ile230 and Asp231 and had a Vina score of −4.3 kcal/mol. Kaempferol therefore showed a more extensive interaction pattern and a more favorable predicted binding score than NAC. Although molecular docking does not establish direct binding or enzymatic activation or inhibition, these findings suggest that kaempferol may have a greater predicted binding affinity for SIRT3 than NAC.

## 4. Discussion

Oxidative stress is increasingly recognized as a major contributor to neuronal dysfunction because neurons have high metabolic demands, substantial oxygen consumption, and relatively limited antioxidant capacity to maintain redox homeostasis under sustained oxidative challenge [20]. In cellular models, exposure to H_2_O_2_ provides a useful means of reproducing several features of oxidative injury, including excessive ROS accumulation, lipid peroxidation, depletion of antioxidant defenses, and subsequent loss of cell viability [13,14,16,21]. The SH-SY5Y cell model is therefore widely used to investigate oxidative stress-induced neuronal injury and evaluate potential neuroprotective compounds. Previous studies have shown that H_2_O_2_ exposure in SH-SY5Y cells increases ROS production, alters antioxidant defenses, and promotes cellular injury, whereas antioxidant or phenolic compounds can attenuate these effects [13,14,18]. In the present study, we examined whether KAE, a dietary flavonol, and NAC, a cysteine donor and glutathione precursor, could provide greater protection when used in combination. The findings indicate that their effects were strongly dependent on the relative concentrations of the two compounds. More importantly, the combination produced a coordinated improvement in cellular antioxidant capacity and was associated with increased SIRT3, FOXO3a, and SOD2 protein levels, together with improved glutathione redox homeostasis.

An important initial consideration in combination studies is whether the concentrations used exert intrinsic cytotoxicity. KAE was well tolerated by SH-SY5Y cells up to 32 μM, whereas concentrations of 64 μM and above significantly reduced cell viability. Similarly, NAC maintained cell viability up to 4 mM but became cytotoxic at 8 mM and above. This concentration-dependent response is consistent with the broader observation that antioxidant compounds do not necessarily remain beneficial as their concentrations increase. Flavonoids can exhibit concentration-dependent cytotoxicity depending on cell type, exposure conditions, and cellular redox status, while high concentrations of NAC may disturb intracellular redox balance rather than simply providing additional antioxidant protection. Previous work in SH-SY5Y cells has demonstrated that NAC can protect against oxidative injury, supporting its use as a redox-modulating agent in this model [22]. The present cytotoxicity data therefore defined an experimental boundary for subsequent combination experiments, allowing the interaction between KAE and NAC to be evaluated within concentration ranges that did not significantly reduce basal cell viability.

The concentration-matrix analysis showed that the interaction between KAE and NAC depended on the concentrations tested. The interaction between KAE and NAC varied across the concentration matrix: combinations with lower NAC concentrations (0.25–1 mM) were predominantly antagonistic, whereas combinations containing 2–4 mM NAC showed additive or synergistic effects. The strongest CI-defined synergy was observed with 16 μM KAE and 2 mM NAC (CI = 0.10). These findings indicate that the interaction between KAE and NAC varied across concentration combinations, rather than producing a uniformly synergistic response. One possible explanation is that the contribution of NAC to cellular protection depends on its concentration, while KAE may contribute additional biological effects through partially distinct mechanisms. However, the present data do not establish the specific mechanisms responsible for the observed concentration-dependent interactions. Such concentration-dependent interactions are consistent with the concept that combination therapy can produce enhanced effects when agents act through complementary rather than identical mechanisms [12]. Previous work in SH-SY5Y cells also showed that combining NAC with another protective compound produced greater cytoprotection than either agent alone, supporting the potential value of multi-target approaches to oxidative neuronal injury [23].

The SynergyFinder analysis provided additional insight into the interaction between KAE and NAC. Positive mean synergy scores were obtained using all four reference models, although the magnitude and statistical significance differed among models. Loewe and HSA analyses reached statistical significance, whereas ZIP and Bliss did not. This lack of consensus indicates that the evidence for synergy depends on the reference model used and does not support a conclusion of broadly consistent synergy across the concentration matrix. Because the four models are based on different assumptions about drug interactions, their results should be interpreted according to their respective reference frameworks rather than treated as interchangeable measures of synergy. Although the HSA model yielded the highest numerical mean synergy score, this value should not be interpreted as demonstrating a stronger synergistic effect than that indicated by the other models. Taken together with the CI analysis, these findings support a model- and concentration-dependent interaction, with evidence of synergy under some analytical frameworks and concentration conditions rather than a uniformly synergistic response across the entire matrix. This concentration dependence also provides a rationale for investigating the selected combination in subsequent mechanistic experiments without assuming that increasing either compound individually would necessarily improve protection.

The functional relevance of this interaction became clearer under H_2_O_2_-induced oxidative stress. H_2_O_2_ markedly decreased SH-SY5Y cell viability, whereas KAE, NAC, and particularly their combination (KNAC) significantly restored cell survival. The protective effect of KAE is consistent with previous reports showing that kaempferol-related flavonols can attenuate H_2_O_2_-induced injury in SH-SY5Y cells [24,25]. Likewise, NAC has long been recognized as a protective agent in SH-SY5Y cells exposed to oxidative stress, in part through its ability to support intracellular glutathione metabolism [26,27]. However, the significantly greater preservation of cell viability with KNAC than with either treatment alone indicates an enhanced cytoprotective response under H_2_O_2_-induced oxidative stress. This greater protective effect does not, by itself, establish synergy, which must be interpreted in the context of the CI and SynergyFinder analyses.

The reduction in CM-H_2_DCFDA fluorescence and MDA levels provides further evidence of the protective effects of KAE and NAC against H_2_O_2_-induced oxidative stress. H_2_O_2_ exposure produced a pronounced increase in CM-H_2_DCFDA fluorescence and MDA, indicating increased intracellular oxidant-sensitive signals and downstream lipid oxidative damage. KAE and NAC each significantly reduced these parameters, while KNAC produced a further reduction compared with either compound alone. These observations agree with previous studies in SH-SY5Y cells in which oxidative injury was accompanied by increased ROS production and lipid peroxidation and was attenuated by phenolic antioxidants or NAC [22,24,25]. CM-H_2_DCFDA fluorescence reflects oxidant-sensitive signals, whereas MDA reflects oxidative damage to membrane lipids. The simultaneous reduction in both endpoints is therefore consistent with attenuation of oxidative stress-associated cellular damage. However, CM-H_2_DCFDA fluorescence does not directly quantify intracellular ROS or identify specific ROS.

The antioxidant enzyme data are consistent with differential contributions of KAE and NAC to cellular antioxidant defense. H_2_O_2_ significantly reduced SOD and CAT activities, consistent with disruption of the enzymatic antioxidant defense system during oxidative stress [14,28]. KAE restored SOD activity, whereas NAC alone did not significantly alter SOD. More notably, KNAC produced a greater increase in SOD activity than either treatment alone. CAT showed an even more selective pattern, with only KNAC significantly restoring activity. These findings suggest that the enhanced protection produced by KNAC is not attributable simply to a generalized increase in antioxidant enzymes. Rather, the combination appears to improve different components of the antioxidant network to different extents. Such coordinated regulation may be advantageous under H_2_O_2_ challenge because SOD and CAT occupy sequential positions in ROS detoxification, with SOD converting superoxide to H_2_O_2_ and CAT subsequently contributing to H_2_O_2_ removal.

GSH-Px displayed a somewhat different response. KAE, NAC, and KNAC all significantly increased GSH-Px activity relative to the H_2_O_2_ group, indicating that this enzyme was responsive to each treatment. Although KNAC produced the numerically highest activity, the difference from either single treatment was not statistically significant. This finding prevents an overly broad conclusion that every component of the antioxidant enzyme system was synergistically enhanced. Instead, the data indicate selective enhancement, with the most pronounced combination effects occurring for SOD and CAT, while GSH-Px was restored by both individual compounds and the combination. Such differential regulation is biologically plausible because antioxidant enzymes are controlled by partially overlapping but distinct regulatory systems [29,30].

The glutathione findings provide a complementary explanation for the particularly strong contribution of NAC to the combined response. GSH is a major intracellular redox buffer and an essential substrate for glutathione-dependent peroxide detoxification. During oxidative stress, GSH is consumed during peroxide reduction, resulting in increased GSSG and a decline in the GSH/GSSG ratio [31,32]. Consistent with this established response, H_2_O_2_ exposure significantly decreased total glutathione and GSH, increased GSSG, and markedly reduced the GSH/GSSG ratio. NAC restored this system, significantly increasing total glutathione and GSH while reducing GSSG. These observations agree with the established role of NAC as a precursor that supplies cysteine for glutathione synthesis and with previous observations that NAC protects SH-SY5Y cells from oxidative stress through modulation of cellular redox status [22,26,33].

The response to KAE alone was notably different. KAE did not significantly restore total glutathione or GSH, although it reduced GSSG and improved the GSH/GSSG ratio. This distinction suggests that KAE may help preserve the redox environment without substantially increasing the cellular glutathione pool. In contrast, NAC directly supports the glutathione system by increasing cysteine availability, providing a biochemical basis for its stronger effect on GSH replenishment. When combined, these complementary properties became particularly apparent: KNAC produced the highest GSH level, the lowest GSSG level, and the highest GSH/GSSG ratio among the treatment groups. The greater response of the combination than either KAE or NAC alone indicates that restoration of glutathione availability and maintenance of redox balance may contribute to the enhanced cytoprotection.

The molecular findings provide a further layer of explanation for these biochemical changes. SIRT3 is a mitochondrial NAD^+^-dependent deacetylase that has an important role in maintaining mitochondrial redox homeostasis. A well-established SIRT3-FOXO3 pathway links mitochondrial stress sensing with the regulation of antioxidant and mitochondrial quality-control responses. SIRT3-mediated deacetylation of FOXO3 promotes mitochondrial homeostasis and increases cellular resistance to oxidative damage [29,34,35]. In neuronal models, disruption of SIRT3 has also been associated with increased oxidative stress, altered SOD2 regulation, and mitochondrial dysfunction [36,37]. In the present study, H_2_O_2_ significantly reduced SIRT3 level, accompanied by reductions in FOXO3a and SOD2. These coordinated changes are consistent with an association between oxidative stress and alterations in proteins involved in mitochondrial antioxidant defense.

KAE and KNAC significantly restored SIRT3 expression, whereas NAC alone did not. KNAC produced significantly higher SIRT3 expression than either single treatment. A similar pattern was observed for FOXO3a and SOD2, with KAE and KNAC restoring their expression while NAC alone failed to reach significance. These findings suggest that KAE may provide a regulatory component of the combination distinct from the direct glutathione-supporting function of NAC. The results are also consistent with emerging evidence that kaempferol can modulate SIRT3-associated antioxidant signaling [38,39]. Recent work in oxidative-stress models has linked kaempferol treatment with increased SIRT3 and altered FOXO3a/SOD2 signaling, although such evidence has primarily been obtained outside the SH-SY5Y neuronal model [38,40]. Thus, the present findings extend the current literature by demonstrating that KAE and KNAC treatment was associated with increased SIRT3, FOXO3a, and SOD2 protein levels in the context of H_2_O_2_-induced oxidative stress in SH-SY5Y cells. However, these findings do not establish that KAE directly activates this pathway or that the pathway is required for the cytoprotective effects of KNAC.

The relationship between SIRT3, FOXO3a, and SOD2 is particularly relevant to interpretation of the present results. SIRT3 can regulate FOXO3 through deacetylation, while FOXO3 contributes to transcriptional control of antioxidant genes, including SOD2. SOD2 is located within mitochondria and represents a major defense against mitochondrial superoxide. Therefore, the simultaneous restoration of SIRT3, FOXO3a, and SOD2 following KNAC treatment provides a biologically coherent link between the molecular findings, reduced CM-H_2_DCFDA fluorescence, and restoration of SOD activity. Importantly, however, the present Western blot data demonstrate association rather than causality. Because SIRT3 enzymatic activity, FOXO3a acetylation or nuclear localization, and SOD2 enzymatic activity were not directly examined, the data do not establish that KNAC protects SH-SY5Y cells specifically through activation of a linear SIRT3/FOXO3a/SOD2 pathway. Overall, the protein-expression data are consistent with an association between KNAC treatment and mitochondrial antioxidant defense; whether this pathway is required for cytoprotection remains unknown.

The docking analysis provides a further, although preliminary, mechanistic clue regarding the contribution of KAE. KAE showed a more favorable predicted docking score for SIRT3 than NAC (−8.2 versus −4.3 kcal/mol) and was predicted to interact with a broader set of residues, including Pro155, Phe180, Leu199, Asn229, Ile230, and His248. The predicted aromatic, hydrophobic, and hydrogen-bond interactions suggest that KAE may interact more extensively with the SIRT3 binding pocket than NAC. This observation is consistent with the experimental finding that KAE, but not NAC, significantly increased SIRT3 expression. However, molecular docking cannot establish direct KAE–SIRT3 binding, SIRT3 enzymatic activation, or a causal role for SIRT3 in mediating the antioxidant effects of KAE or KNAC. Therefore, the present findings support a potential association between KAE and SIRT3, while direct binding and functional regulation of SIRT3 require further validation using biochemical binding assays, SIRT3 activity measurements, or loss-of-function approaches.

Taken together, KAE appears to contribute more strongly to the protein-regulatory component, particularly the SIRT3/FOXO3a/SOD2 axis, whereas NAC provides a metabolic contribution to glutathione replenishment. KNAC therefore engages both enzyme-regulatory and substrate-dependent aspects of cellular antioxidant defense, providing a mechanistic framework for the enhanced responses observed across multiple endpoints.

An additional point emerging from the present study is that the interaction between KAE and NAC cannot be inferred simply from the concentration of either compound. The strongest CI-defined interaction occurred at 16 μM KAE plus 2 mM NAC (CI = 0.10), corresponding to a KAE:NAC molar ratio of 1:125, whereas antagonistic interactions were observed at lower NAC concentrations. Together with the SynergyFinder results, these findings indicate that the interaction depends on both concentration and the reference model used to define synergy. Although the selected combination produced enhanced cytoprotective effects in the present cellular model, the available evidence does not establish that the combination is broadly synergistic across all concentration conditions. The findings therefore support further investigation of KAE–NAC combinations as a potential food-derived or nutraceutical strategy for maintaining neuronal redox homeostasis, while recognizing that the present findings remain at the cellular level.

An important consideration for the translational relevance of these findings is the concentration of NAC used in the present in vitro experiments. Although NAC concentrations of 0.25–4 mM were selected based on the cytotoxicity assessment in SH-SY5Y cells, the concentrations applied directly to cultured cells cannot be assumed to reflect those achievable in human plasma or neuronal tissue following administration. Oral NAC has low bioavailability because of extensive intestinal and hepatic metabolism, while its ability to cross the blood–brain barrier remains dependent on the route of administration and is not fully established [41,42]. Similarly, kaempferol undergoes absorption and extensive metabolism, and its systemic bioavailability depends on its chemical form and dietary source [43]. Accordingly, the protective effects observed at millimolar NAC concentrations, including the interaction identified at 2 mM NAC, should be interpreted within the context of this in vitro model rather than as evidence that comparable exposures can be achieved in the human brain. Further pharmacokinetic studies and validation in physiologically relevant models are therefore needed to determine whether exposures sufficient to reproduce the observed cytoprotective effects can be achieved in vivo.

Several limitations should nevertheless be acknowledged. First, the study was conducted in an undifferentiated SH-SY5Y cell model, and the response of differentiated neurons or primary human neuronal cells may differ. Second, although the coordinated changes in SIRT3, FOXO3a, and SOD2 suggest an association with cellular antioxidant defense, the absence of functional experiments, such as SIRT3 inhibition or knockdown, prevents us from determining whether this pathway is required for the cytoprotective effects of KNAC. In addition, SIRT3 enzymatic activity, FOXO3a acetylation or subcellular localization, and SOD2 enzymatic activity were not directly assessed. Therefore, the proposed pathway should be considered a potential mechanism that requires further experimental validation. Third, the docking analysis provides structural predictions rather than experimental evidence of direct KAE–SIRT3 binding. Fourth, cell viability was assessed using the MTT assay. Although cell-free controls were included to evaluate potential interference by KAE and NAC, potential interference by H_2_O_2_ was not independently assessed in a cell-free system, as H_2_O_2_ was used at a fixed concentration to induce oxidative stress. Furthermore, the absence of an independent viability assay means that assay-related effects cannot be completely excluded. In addition, CM-H_2_DCFDA provides a general oxidant-sensitive signal rather than a specific measure of individual ROS, and the absence of a complementary assay limits the specificity of these findings. Fifth, the pharmacokinetics and systemic and brain exposures of KAE and NAC were not assessed. Therefore, it remains uncertain whether the concentrations associated with cytoprotection in vitro can be achieved in vivo, particularly in neuronal tissue. Finally, H_2_O_2_-induced oxidative stress represents a simplified model and does not reproduce the complex cellular interactions, mitochondrial alterations, and chronic stress conditions associated with human neurodegenerative diseases. These limitations highlight the need for further studies to validate the proposed mechanisms, corroborate the oxidative stress-related findings using complementary methods, and establish the mechanistic and translational relevance of the KAE–NAC combination.

## 5. Conclusions

Overall, this study provides evidence that KAE and NAC produce enhanced cytoprotective effects against H_2_O_2_-induced oxidative injury in SH-SY5Y cells, with the evidence for synergy varying according to the concentration combination and reference model used for analysis. The enhanced protection was associated with reduced CM-H_2_DCFDA fluorescence and MDA level, improved antioxidant enzyme activities and glutathione redox balance, and increased protein levels of SIRT3, FOXO3a, and SOD2. These complementary responses suggest that KAE and NAC may contribute to cellular antioxidant defense through distinct but potentially complementary processes. The increased levels of SIRT3, FOXO3a, and SOD2 suggest an association with mitochondrial antioxidant defense, whereas NAC may support glutathione-dependent redox buffering. However, the causal involvement of the SIRT3/FOXO3a/SOD2 pathway in the cytoprotective effects of KNAC remains to be established. The predicted interaction between KAE and SIRT3 provides a hypothesis for further investigation; however, direct binding and functional regulation of SIRT3 have not been experimentally confirmed. Collectively, these findings support further investigation of the KAE–NAC combination as a potential strategy for enhancing cellular antioxidant defenses by combining a food-derived KAE with NAC, a glutathione precursor. Further studies are needed to establish the causal mechanisms, validate the predicted interaction, and determine the relevance of these findings in more physiologically representative models.

## Figures and Tables

**Table 1:** Effects of KNAC on glutathione redox status.

Groups	Total Glutathione (nmol/mg Protein)	GSH (nmol/mg Protein)	GSSG (nmol/mg Protein)	GSH/GSSG
Control	94.29 ± 1.52	89.37 ± 1.21	4.92 ± 0.31	18.26 ± 0.87
H_2_O_2_ + vehicle	65.01 ± 2.27 **	49.01 ± 1.81 ***	16.00 ± 0.47 ***	3.06 ± 0.03 ***
H_2_O_2_ + KAE	68.02 ± 7.07 **^, †^	57.67 ± 7.08 ***^, †^	10.35 ± 0.11 ***^, ###, †††^	5.57 ± 0.70 ***^, #, †††^
H_2_O_2_ + NAC	81.95 ± 1.05 ^#^	74.22 ± 0.96 ^##^	7.73 ± 0.15 ***^, ###^	9.61 ± 0.17 ***^, ###, †^
H_2_O_2_ + KNAC	89.59 ± 1.70 ^##^	82.73 ± 1.63 ^###^	6.86 ± 0.08 *^, ###^	12.06 ± 0.14 **^, ###^

Data are presented as mean ± SEM from three independent experiments. *, **, and ***, *p* < 0.05, *p* < 0.01, and *p* < 0.001, respectively, compared with the control group; #, ##, and ###, *p* < 0.05, *p* < 0.01, and *p* < 0.001, respectively, compared with the H_2_O_2_ + vehicle group; †, and †††, *p* < 0.05, and *p* < 0.001, respectively, compared with the KNAC group. KAE, kaempferol; NAC, N-acetylcysteine; KNAC, the combination of KAE and NAC; GSH, reduced glutathione; GSSG, oxidized glutathione.

**Table 2:** Predicted interactions of KAE and NAC with human SIRT3.

Ligand	Vina Score (kcal/mol)	SIRT3 Residue	Interaction Type
KAE	−8.2	Pro155	Conventional hydrogen bond
		Phe180	π–π interaction
		Leu199	π–alkyl
		Asn229	Conventional hydrogen bond
		Ile230	π–alkyl
		His248	π–σ/aromatic interaction
NAC	−4.3	Ile230	Conventional hydrogen bond
		Asp231	Conventional hydrogen bond

KAE, kaempferol; NAC, N-acetylcysteine; SIRT3, sirtuin 3.

## Data Availability

The original contributions presented in this study are included in the article/Supplementary Materials. Further inquiries can be directed to the corresponding author.

## Supplementary Materials

The following supporting information can be downloaded at: https://www.mdpi.com/article/10.3390/foods15213865/s1, Figure S1: Evaluation of potential MTT assay interference by kaempferol (KAE) and N-acetylcysteine (NAC) using cell-free controls. (A) Change in cell-free MTT signal (%) in the presence of KAE. (B) Change in cell-free MTT signal (%) in the presence of NAC. The results are expressed as percentage changes relative to the corresponding vehicle controls. * *p* < 0.05 compared with the control in panel A; # *p* < 0.05 compared with the control in panel B; Figure S2: Original, unprocessed, and uncropped full-length membrane images from Western blot analysis of SIRT3 expression, with β-actin as the internal loading control. The experimental groups were as follows: (1) control, (2) H_2_O_2_ + vehicle, (3) H_2_O_2_ + KAE, (4) H_2_O_2_ + NAC, and (5) H_2_O_2_ + KNAC. H_2_O_2_, hydrogen peroxide; KAE, kaempferol; NAC, N-acetylcysteine; KNAC, the combination of kaempferol and Nacetylcysteine; SIRT3, sirtuin 3; β-actin, beta-actin; Figure S3: Original, unprocessed, and uncropped full-length membrane images from Western blot analysis of FOXO3a and SOD2 expression, with β-actin as the internal loading control. The experimental groups were as follows: (1) control, (2) H_2_O_2_ + vehicle, (3) H_2_O_2_ + KAE, (4) H_2_O_2_ + NAC, and (5) H_2_O_2_ + KNAC. H_2_O_2_, hydrogen peroxide; KAE, kaempferol; NAC, N-acetylcysteine; KNAC, the combination of kaempferol and N-acetylcysteine; FOXO3a, forkhead box O3a; SOD2, superoxide dismutase 2; β-actin, beta-actin; Figure S4: Representative original, unprocessed phase-contrast microscopic images of SH-SY5Y cells from the five experimental groups at 10× magnification. The experimental groups were as follows: (1) control, (2) H_2_O_2_ + vehicle, (3) H_2_O_2_ + KAE, (4) H_2_O_2_ + NAC, and (5) H_2_O_2_ + KNAC. H_2_O_2_, hydrogen peroxide; KAE, kaempferol; NAC, N-acetylcysteine; KNAC, the combination of kaempferol and N-acetylcysteine.
