# Supplementary material for: Combined Antioxidant Effects of Kaempferol and N-Acetylcysteine on H2O2-Induced Oxidative Stress in SH-SY5Y Cells
Source: Foods. 2026 Oct 28;15(21):3865. doi: 10.3390/foods15213865 (PMC13763044; doi:10.3390/foods15213865)
Supplement: Supplementary file 1 [file foods-15-03865-s001.zip › foods-4601526-supplementary.pdf]

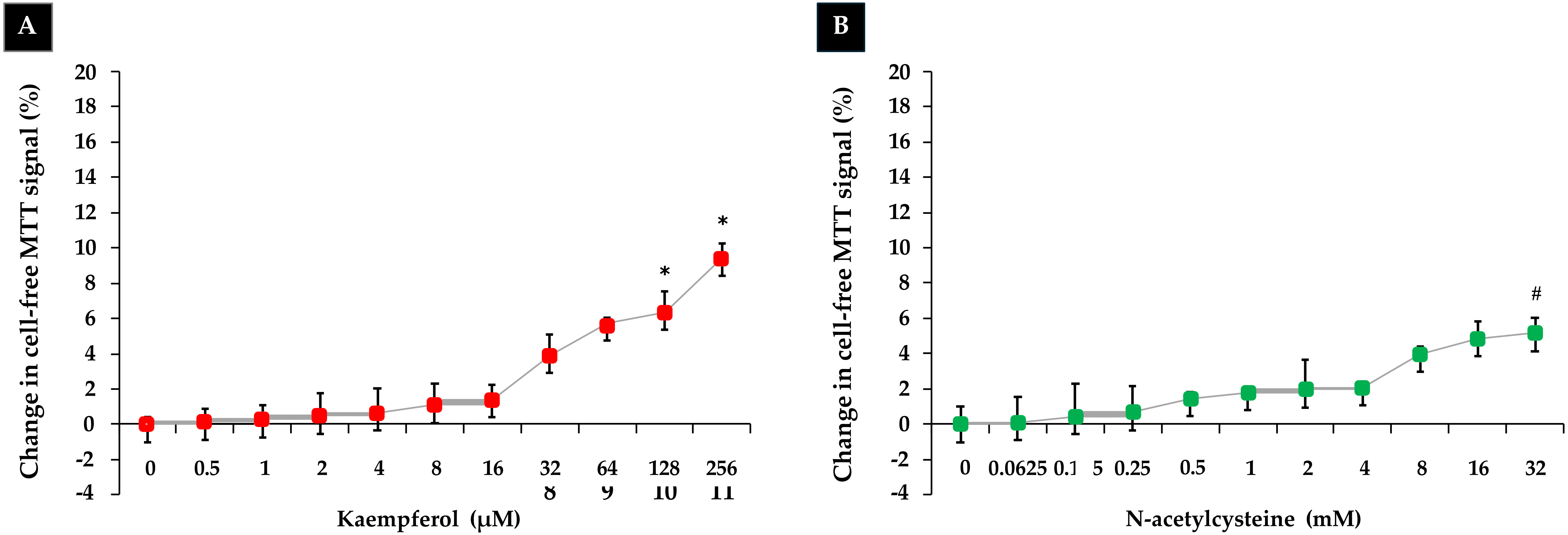

Figure S1: Evaluation of potential MTT assay interference by kaempferol (KAE) and N-acetylcysteine (NAC) using cell-free controls. (**A**) Change in cell-free MTT signal (%) in the presence of KAE. (**B**) Change in cell-free MTT signal (%) in the presence of NAC. The results are expressed as percentage changes relative to the corresponding vehicle controls. * $p < 0.05$ compared with the control in panel (**A**); # $p < 0.05$ compared with the control in panel (**B**).

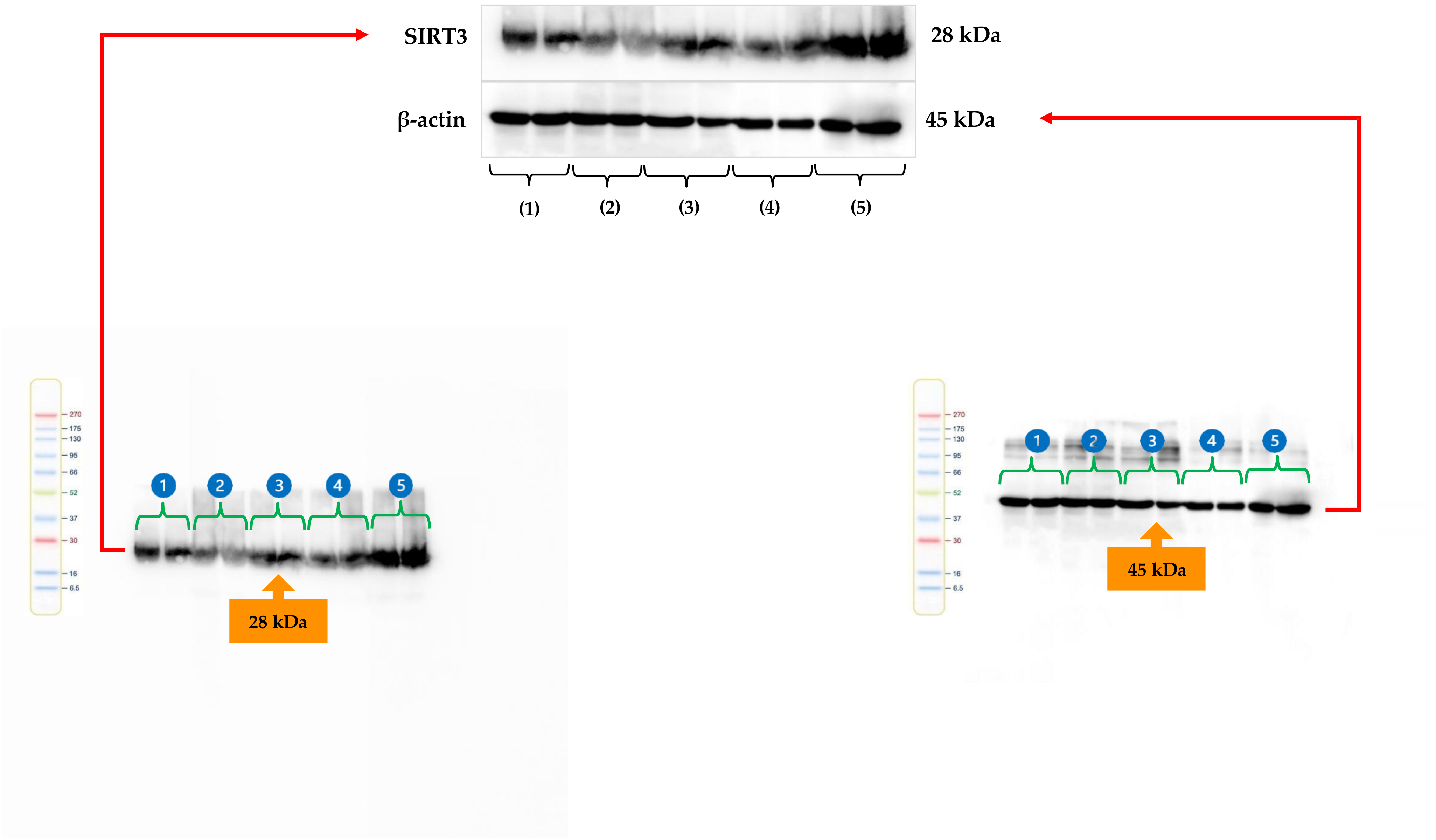

Figure S2: Original, unprocessed, and uncropped full-length membrane images from Western blot analysis of SIRT3 expression, with β-actin as the internal loading control. The experimental groups were as follows: (1) control, (2) H_2O_2 + vehicle, (3) H_2O_2 + KAE, (4) H_2O_2 + NAC, and (5) H_2O_2 + KNAC. H_2O_2, hydrogen peroxide; KAE, kaempferol; NAC, N-acetylcysteine; KNAC, the combination of kaempferol and N-acetylcysteine; SIRT3, sirtuin 3; β-actin, beta-actin.

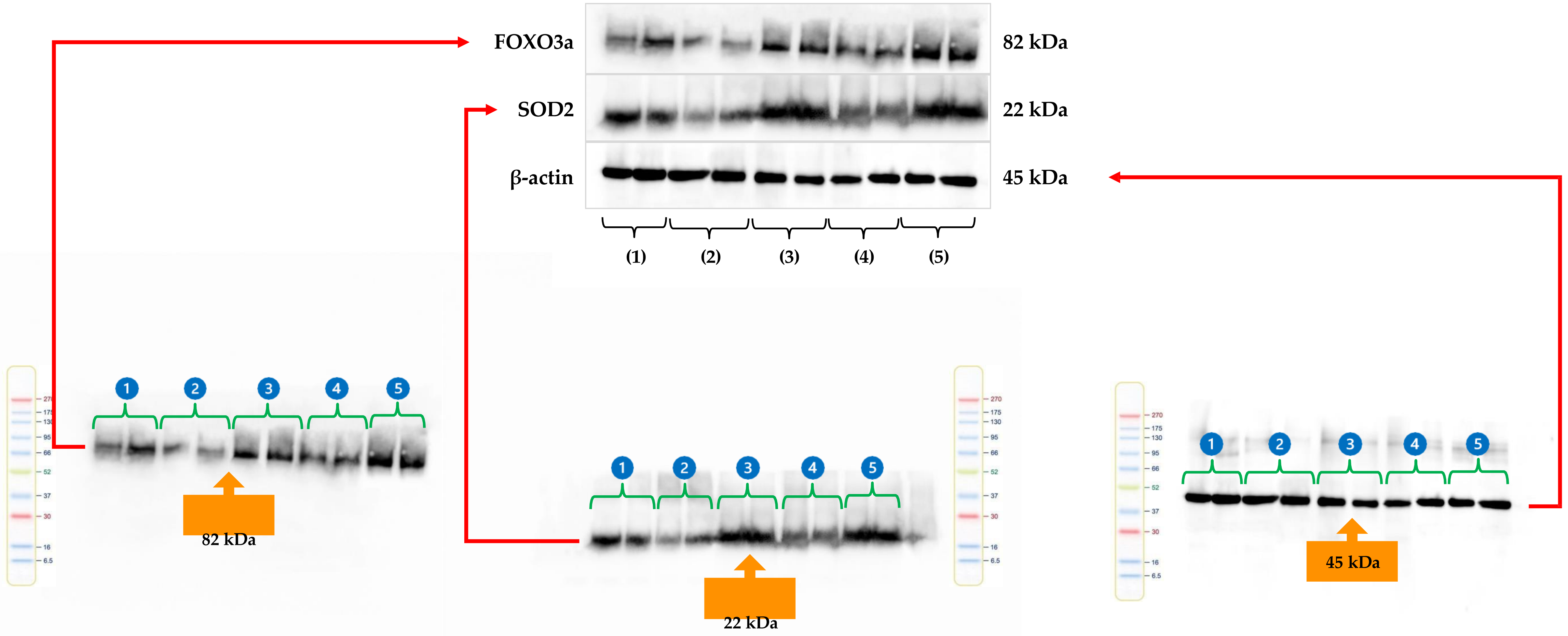

Figure S3: Original, unprocessed, and uncropped full-length membrane images from Western blot analysis of FOXO3a and SOD2 expression, with β-actin as the internal loading control. The experimental groups were as follows: (1) control, (2) H_2O_2 + vehicle, (3) H_2O_2 + KAE, (4) H_2O_2 + NAC, and (5) H_2O_2 + KNAC. H_2O_2, hydrogen peroxide; KAE, kaempferol; NAC, N-acetylcysteine; KNAC, the combination of kaempferol and N-

acetylcysteine; FOXO3a, forkhead box O3a; SOD2, superoxide dismutase 2; β-actin, beta-actin.

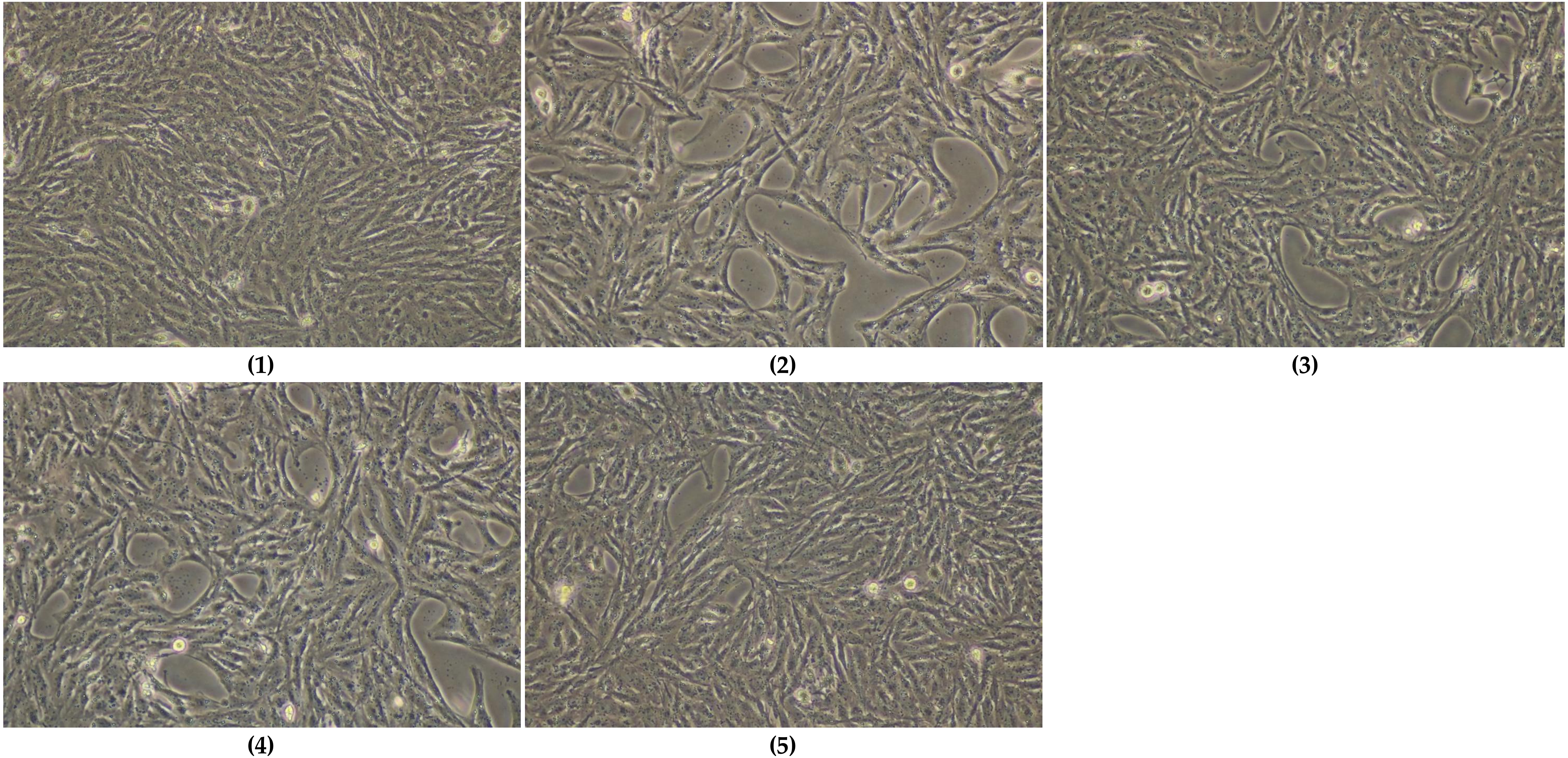

Figure S4: Representative original, unprocessed phase-contrast microscopic images of SH-SY5Y cells from the five experimental groups at 10× magnification. The experimental groups were as follows: (1) control, (2) H_2O_2 + vehicle, (3) H_2O_2 + KAE, (4) H_2O_2 + NAC, and (5) H_2O_2 + KNAC. H_2O_2, hydrogen peroxide; KAE, kaempferol; NAC, N-acetylcysteine; KNAC, the combination of kaempferol and N-acetylcysteine.
